# The *Musa troglodytarum* L. genome provides insights into the mechanism of non-climacteric behaviour and enrichment of carotenoids

**DOI:** 10.1186/s12915-022-01391-3

**Published:** 2022-08-24

**Authors:** Zhiying Li, Jiabin Wang, Yunliu Fu, Yonglin Jing, Bilan Huang, Ying Chen, Qinglong Wang, Xiao Bing Wang, Chunyang Meng, Qingquan Yang, Li Xu

**Affiliations:** 1 Institute of Tropical Crop Genetic Resources, Chinese Academy of Tropical Agricultural Sciences, Danzhou, 571737 Hainan China; 2 Ministry of Agriculture Key Laboratory of Crop Gene Resources and Germplasm Enhancement in Southern China, Danzhou, 571737 Hainan China; 3 Hainan Province Key Laboratory of Tropical Crops Germplasm Resources Genetic Improvement and Innovation, Danzhou, 571737 Hainan China; 4 National Gene Bank of Tropical Crops, Danzhou, 571700 Hainan China; 5 College of Horticulture and Landscape Architecture, Hainan University, Haikou, 570228 China

**Keywords:** Banana, Chromosome-level genome, Carotenoids, Non-climacteric behaviour

## Abstract

**Background:**

Karat (*Musa troglodytarum* L.) is an autotriploid Fe’i banana of the Australimusa section. Karat was domesticated independently in the Pacific region, and karat fruit are characterized by a pink sap, a deep yellow-orange flesh colour, and an abundance of β-carotene. Karat fruit showed non-climacteric behaviour, with an approximately 215-day bunch filling time. These features make karat a valuable genetic resource for studying the mechanisms underlying fruit development and ripening and carotenoid biosynthesis.

**Results:**

Here, we report the genome of *M. troglodytarum*, which has a total length of 603 Mb and contains 37,577 predicted protein-coding genes. After divergence from the most recent common ancestors, *M. troglodytarum* (T genome) has experienced fusion of ancestral chromosomes 8 and 9 and multiple translocations and inversions, unlike the high synteny with few rearrangements found among *M. schizocarpa* (S genome), *M. acuminata* (A genome) and *M. balbisiana* (B genome). Genome microsynteny analysis showed that the triplication of *MtSSUIIs* due to chromosome rearrangement may lead to the accumulation of carotenoids and ABA in the fruit. The expression of duplicated *MtCCD4*s is repressed during ripening, leading to the accumulation of α-carotene, β-carotene and phytoene. Due to a long terminal repeat (LTR)-like fragment insertion upstream of *MtERF11*, karat cannot produce large amounts of ethylene but can produce ABA during ripening. These lead to non-climacteric behaviour and prolonged shelf-life, which contributes to an enrichment of carotenoids and riboflavin.

**Conclusions:**

The high-quality genome of *M. troglodytarum* revealed the genomic basis of non-climacteric behaviour and enrichment of carotenoids, riboflavin, flavonoids and free galactose and provides valuable resources for further research on banana domestication and breeding and the improvement of nutritional and bioactive qualities.

**Supplementary Information:**

The online version contains supplementary material available at 10.1186/s12915-022-01391-3.

## Background

Bananas (*Musa* spp.) are among the most favoured fruits worldwide and are important staple foods for people in some tropical and subtropical countries [1]. According to their distinct genetic backgrounds, four genomes are represented within *Musa* spp., including *Musa acuminata* (A genome, 2n = 2x = 22), *Musa balbisiana* (B genome, 2n = 2x = 22), *Musa schizocarpa* (S genome, 2n = 2x = 22) and the *Australimusa* species (T genome, 2n = 2x = 20) [2]. Bananas are typically triploids or diploids of subspecies of *Musa acuminata* or of *Musa balbisiana* and *M. acuminata.* Of the seven species of *Australimusa*, there is a distinct group of banana species known as Fe’i banana (*Musa troglodytarum* L.). Fe’i banana plants are characterized by erect bunches and produce fruit that have a deep yellow-orange flesh colour and an abundance of α-carotene and β-carotene [3]. Karat is a cultivar of Fe’i banana distributed on the island of Pohnpei. Karat used to be a traditional weaning food in Pohnpei and has regained popularity due to a campaign that promoted karat to combat vitamin A deficiency (VAD) [4]. Moreover, karat has also been found to be rich in riboflavin (vitamin B2), an essential vitamin for nervous system function and iron utilization [5]. Riboflavin also contributes to the yellow colour of karat flesh.

Carotenoids are a large group of isoprenoids that play essential roles in plants; carotenoids function as pigments in both photosynthesis and light harvesting and serve as substrates for the biosynthesis of strigolactone and abscisic acid (ABA) [6]. The carotenoid biosynthetic pathway has been described in plants [7]. 1-Deoxy-D-xylulose-5-phosphate synthase (*DXS*) is the first and rate-limiting enzyme of the MEP pathway, and phytoene synthase regulates the first step of carotenoid biosynthesis by condensation of geranylgeranyl diphosphate, which is rate-limiting [8, 9]. Both α- and β-carotene are produced through the cyclization of lycopene by ε-lycopene cyclase (*LCYE*) and β-lycopene cyclase (*LCYB*). The pro-vitamin A (PVA) content of Musa germplasms varied from 0 to 85.08 μg/g, with yellow-orange plantains, Papua New Guinea diploids and deep yellow-orange pulp Fe’i bananas have relatively high carotenoid contents [10]. The biosynthesis of carotenoids of the Fe’i cultivar Asupina has been examined, and *MtCCD4* is thought to be the key gene that results in hyperaccumulation in Asupina [11]. *MtPSY2a* cloned from Asupina was successfully used to generate transgenic bananas with high PVA levels [12]. In Cavendish, *MaSPL6* functions as an activator of *MaLCYB1.1*, and *MaLCYB1.2* and plays essential roles in carotenoid accumulation during ripening [13]. High carbohydrate contents were also shown to contribute to the hyperaccumulation of carotenoids in the green mutant of the plantain variety Obubit Ntanga [14].

Fe’i banana fruit are a parthenocarpic edible type [15]. The domestication of Fe’i banana occurred independently of that of plantains and other banana species through parthenocarpy and sterility processes [15]. To date, the genomes of A, B, S and *Musa itinerans* of *Musa* spp. have been published [16–19]. In addition to the latest updated genome of *Musa acuminata* DH PaHang, the genome data of *Musa acuminata* Banksii, Zebrina, and Calcutta 4 were deposited in the banana genome hub [20]. A cross-genus pangenome of banana contains representatives of the *Musa* and *Ensete* genera was presented, including genomic short reads of *Musa troglodytarum L.* ‘Pisang Tongkat Langit’ (tongkat), a cultivar from Eastern Indonesia [21, 22]. However, limited information is available for the *Musa troglodytarum L.* (T) genome, which has restricted the mining and utilization of valuable germplasm and gene resources. In this study, we de novo sequenced the genome of *Musa troglodytarum* L. for the first time, by integrating Oxford Nanopore, PacBio, Illumina and Hi-C sequencing techniques. Karat fruit showed non-climacteric behaviour, with an approximately 215-day bunch filling time. Metabolomic analyses and transcriptome sequencing were carried out to determine candidate genes involved in non-climacteric behaviour and the enrichment of carotenoids and riboflavin. The data from this study will be valuable for further research on improving the nutritional and bioactive qualities of banana fruit, prolonging shelf-life and reducing postharvest crop losses.

## Results

### Assembly of the T genome

**Fig. 1:**
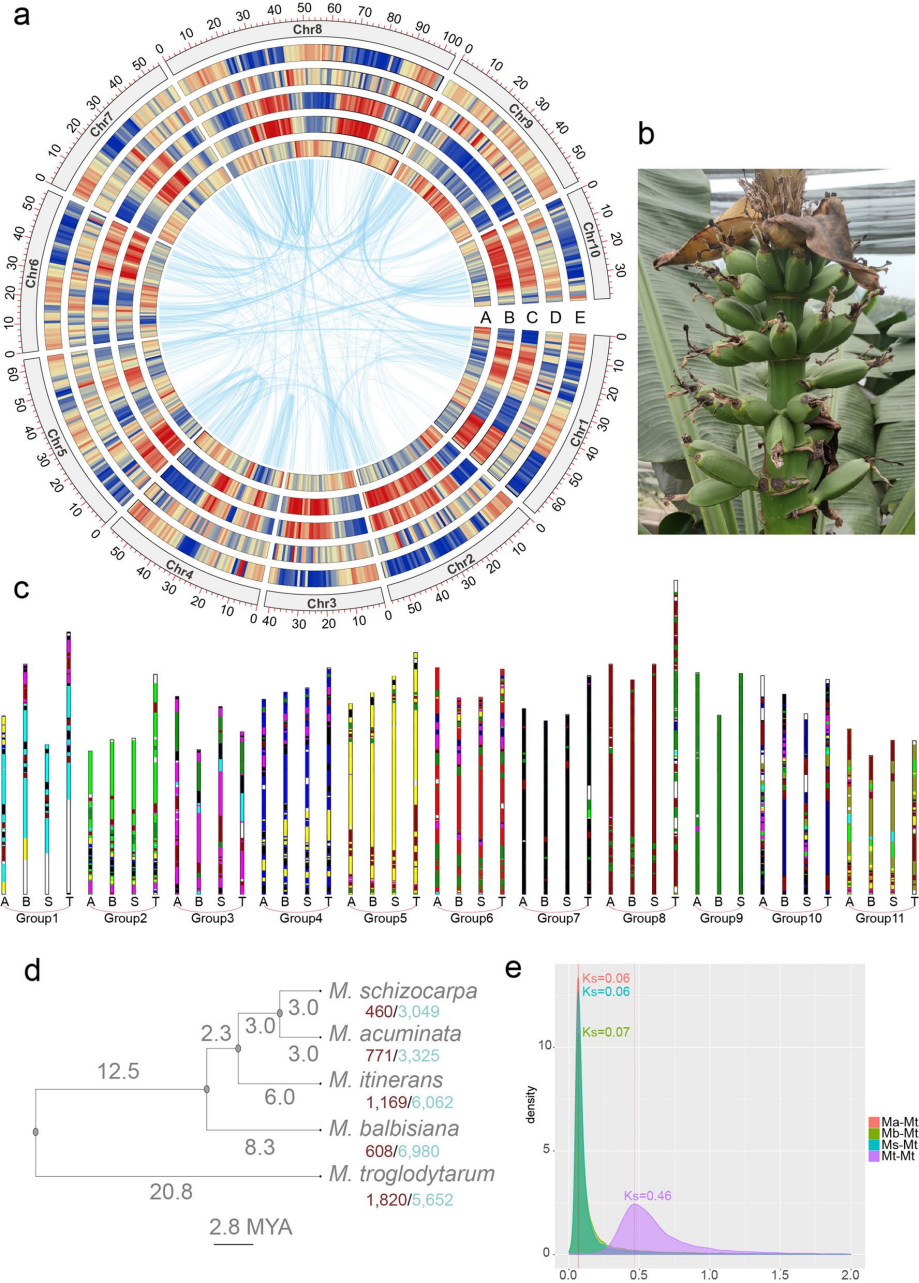
Overview of the T genome. **a** Chromosome overview of the T genome. A, GC content; B, repeat content; C LTR Copia content; D, LTR gypsy content; and E, gene content. **b** Erect fruit bunch of 25 DAF karat, which is pictured at Danzhou, Hainan, China. **c** Ancestor genome analysis and chromosome rearrangements. The bars representing the chromosomes of the A, B, S and T genomes are divided into 11 groups. Each colour presents one of the ancestral chromosomes. Phylogenetic analysis (**d**) and distribution of the 4 dTv distances between gene pairs (**e**) of the A, B, S and T genomes

The chromosomes of karat were fluorescently stained, and the result showed that karat is an autotriploid cultivar with 30 chromosomes (Additional file 1: Fig. S1). According to the genome survey, the T genome is 606~655 Mb in size and has a heterozygosity rate ranging from 1.25% (tongkat, TT) to 1.55% (karat, TTT) (Additional file 1: Fig. S2 and Additional file 1: Table S1-2). For genome sequencing, we generated 42 Gb of Nanopore reads, 6.9 Gb of PacBio reads and 42 Gb of Illumina reads (Additional file 1: Table S3-5). Using NextDenovo and NextPolish, we obtained an assembly with a total length of 918 Mb and contig N50 of 4.9 Mb (Additional file 1: Table S6). After purging haplotigs (Additional file 1: Fig. S3), we obtained 603 Mb contigs (Additional file 1: Table S7), and with 110 Gb of Hi-C reads mapped, the contigs were arranged into 10 chromosomes (Fig. 1a and Additional file 1: Fig. S4). BUSCO analysis showed that 97.7% of the BUSCO genes were assembled (Additional file 1: Table S8). The transcriptomes of leaves, roots, stems and fruits were sequenced for gene annotation. Using Maker2 [23], we predicted 37,577 protein-coding genes (Additional file 1: Table S9). BUSCO analysis showed that 92.5% of the BUSCO genes were predicted (Additional file 1: Table S10). Using eggNOG-mapper [24], we predicted 30,377 protein-coding genes with orthologues, 16,687 genes with GO annotation and 13,105 genes with KEGG annotation. Using RepeatMasker [25], we found that 59.62% of the T genome contained repeat elements (Additional file 1: Table S11). LTR/Gypsy and LTR/Copia accounted for 15.1% and 36.4% of the genome, respectively. As specific marker of the centromeric regions in *M. acuminata* genome [16, 26], Nanica LINE clusters also presented in all chromosomes (Additional file 1: Fig. S5). Using LTR FINDER [27], we identified 3,128 intact LTRs, and analysis of the insertion time showed that there was an LTR insertion burst at 1.47 MYA (Additional file 1: Fig. S6), which occurred before the burst of *M. balbisiana* (0.32 MYA) and after the burst of *M. acuminata* (1.77 MYA). Then, Illumina reads of karat and tongkat were mapped to the T genome. There were 516,884 and 459,137 indels, and 7,716,375 and 7,125,857 single-nucleotide polymorphism (SNP) sites identified in karat and tongkat, respectively (Additional file 1: Table S12 and Additional file 1: Fig. S7).

Using OrthoFinder [28], we identified 8924 single-copy genes and 27,100 orthologous gene sets in the A, B, S, T and *M. itinerans* genomes. There were 7791 genes specific in the T genome. According to the phylogenetic tree generated by OrthoFinder and the divergence time of *M. acuminata* and *M. balbisiana* reported in a previous study [17], we constructed an ultrametric tree showing that *M. troglodytarum* diverged from the ancestor of *M. acuminata*, *M. schizocarpa* and *M. balbisiana* 20.8 MYA (Fig. 1d). According to the 4DTV distances, the peak *Ks* values were approximately 0.06, 0.06, and 0.07 for *M. troglodytarum–M. acuminata*, *M. troglodytarum–M. schizocarpa* and *M. troglodytarum–M. balbisiana,* respectively, where a peak *Ks* value of approximately 0.46 indicated whole-genome duplication (Fig. 1e). Using Café [29], we identified 771, 460, 1820 and 608 expanded gene families and 3325, 3049, 562 and 6980 contracted gene families in the A, S, T and B genomes, respectively. There are 11 gene families with rapid expansions in T genome. GO functional enrichment analysis was conducted to explore the functions T genome-specific genes and rapidly evolving families (Additional file 1: Fig. S8-9 and supplementary Additional file 2: Data 1-2). GO enrichment analysis show that those genes of rapidly evolving gene families were enriched in cell morphogenesis, cell growth, defense response to insect immune system process, defense response to bacterium, defense response to fungus, response to virus etc.

Using MGRA2 [30], we constructed the ancestral genome of the A, B, S and T genomes, which resulted in 11 contiguous ancestral regions (CARs) and 20,056 ordered ancestral genes. For chromosome rearrangement, we constructed bar plots of the A, B, S and T genomes compared to CARs using MCSCAN (Fig. 1c). The ancestor of the A, B, S and T genomes experienced multiple chromosome rearrangements before and after their divergence. Chromosome 1 of the A, B and T genomes experienced translocation after divergence, and chromosomes8 and 9 in the ancestry fused into chromosome 9 of the T genome. A dot plot of the synteny gene blocks between *M. troglodytarum* and *M. acuminata* also indicated the fusion of chromosomes 8 and 9 in the T genome (Additional file 1: Fig. S10-11). *M. troglodytarum* was domesticated independently and diverged from an ancestor of *M. acuminata*, *M. schizocarpa* and *M. balbisiana* 20.8 MYA. Thus, *M. troglodytarum* has experienced multiple translocations and inversions, unlike the high synteny with few rearrangements found among *M. schizocarpa*, *M. acuminata* and *M. balbisiana*.

### Transcriptome and metabolome of the fruit

**Fig. 2:**
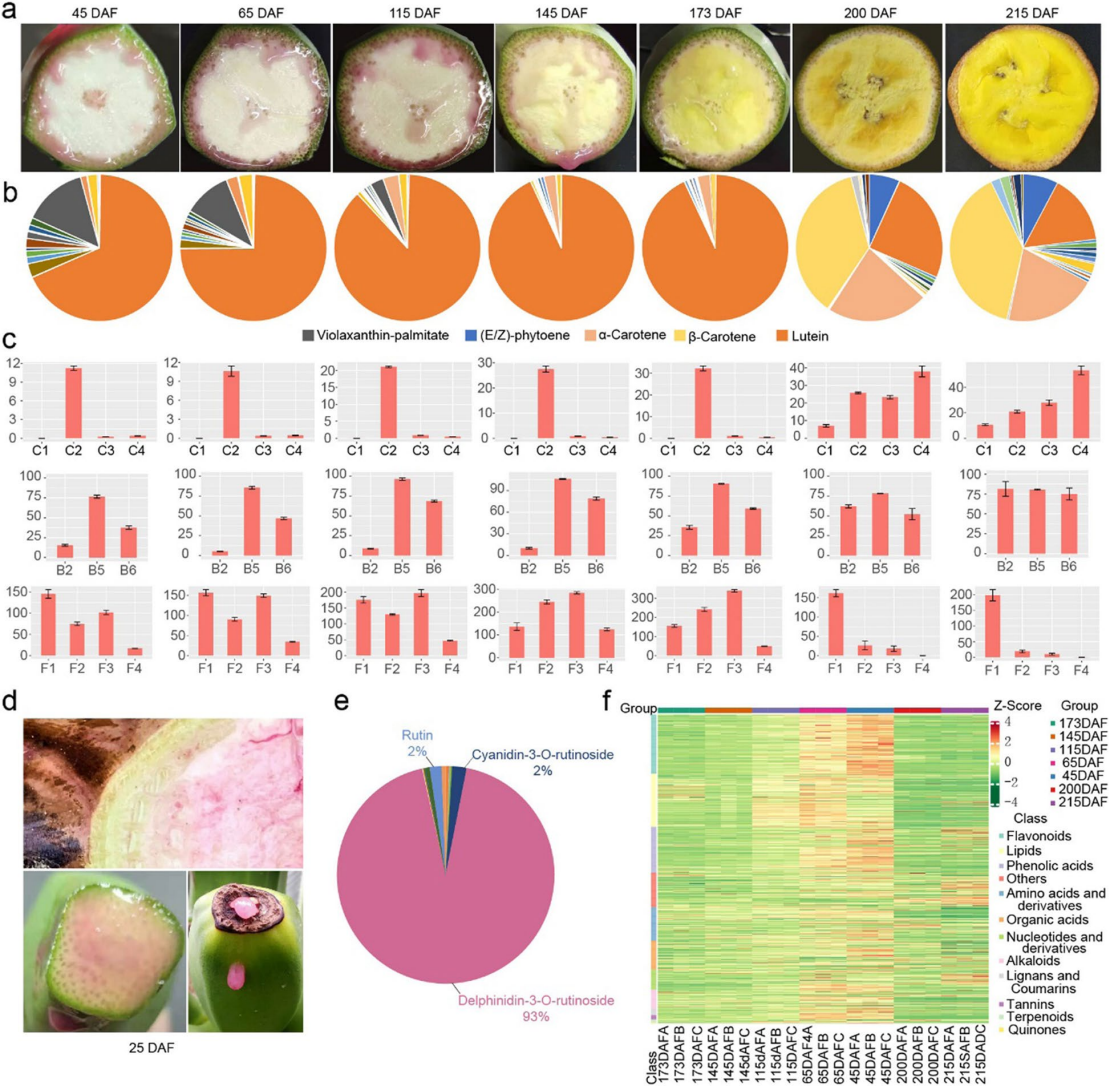
Targeted metabolomics and widely targeted metabolomics analysis of karat pulp at different developmental stages. **a** Transverse section of karat fruit at different developmental stages. Quantitation of carotenoids (**b** and **c**, μg/g) and relative quantification of flavonoids (**c**) and vitamin B (**c**). F1, (E/Z)-phytoene; F2, Lutein; F3, α-Carotene; F4, β-Carotene; B2, riboflavin; B5, D-pantothenic acid; B6, pyridoxine; F1, 4′-hydroxy-5,7-dimethoxyflavanone; F2, epicatechin; F3, myricetin-3-O-rutinoside; and F4, delphinidin-3-O-rutinoside. **d** Pink sap of pesudostem and fruits at 25 DAF. **e** Quantitation of flavonoids in pink sap. **f** Heatmap of metabolites in fruit pulp

To determine the basis of the enrichment of carotenoids and flavonoids and the non-climacteric behaviour of karat, we integrated widely targeted metabolomics and targeted metabolomics data from karat pulp at 25, 45, 65, 115, 145, 173, 200 and 215 days after flowering (DAF) and RNA sequencing (RNA-seq) data from karat pulp at 25, 45, 65, 115, 100, 130, 145, 152, 159, 173, 200 and 208 DAF (Fig. 2a). According to widely targeted metabolomic data, we identified 877 metabolites, including flavonoids, lipids, phenolic acids, amino acids and their derivatives, organic acids, nucleotides and their derivatives, alkaloids, lignin, coumarins, tannins, terpenoids, quinones and others, 768 of them were divided into 5 clusters (Fig. 2f, Additional file 1: Fig. S12 and Additional file 2: Data 3-4). Cluster 2 represents the metabolites that increased during ripening, including alkaloids, amino acids and their derivatives, coumarins, free fatty acids, organic acids, phenolic acids, saccharides and alcohols, vitamins and others.

#### Carotenoids were enriched throughout fruit development

**Fig. 3:**
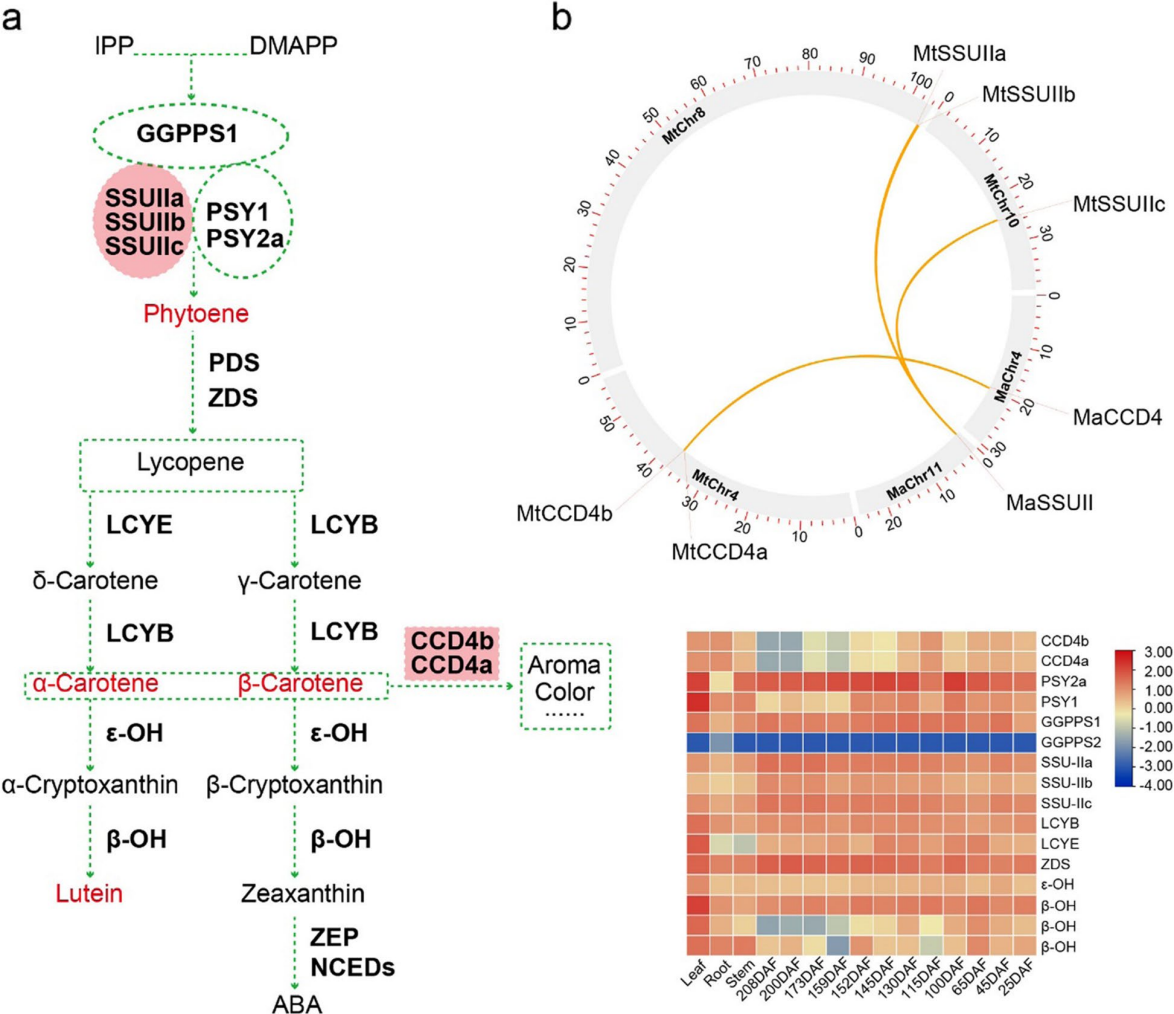
Schematic representation of the carotenoid biosynthesis pathway and duplication of key enzymes in karat. Geranylgeranyl pyrophosphate synthase small subunit (SSU-II) and carotenoid cleavage dioxygenase 4 (CCD4) are duplicated in the T genome compared to the A, B, and S genomes. Phytoene, α-carotene and β-carotene contents were highly enriched in karat pulp. **a** Schematic representation of the vitamin E and carotenoid biosynthesis pathways in karat. Interactions are represented by red dotted lines. IPP, isopentenyl diphosphate; DMAPP, dimethylallyl diphosphate; GGPP, geranylgeranyl pyrophosphate; GGPPS, geranylgeranyl pyrophosphate synthase; PSY, phytoene synthase; PDS, phytoene desaturase; ZDS, ζ-carotene desaturase; LCYB, lycopene β-cyclase; LCYE, lycopene ε-cyclase; β-OH, carotene β-hydroxylase; ε-OH, ε-hydroxylase; ZEP, zeaxanthin epoxidase; and NCED, 9-cis-epoxycarotenoid dioxygenase. **b** Distribution of SSUll and CCD4 genes and the distribution and synteny between the T genome and A genome. **c** Heatmap of carotenoid biosynthesis pathway genes in different tissues

According to the quantification of the karat pulp metabolites, lutein accumulated throughout all the fruit developmental stages, with a content of 10.62–32.25 μg/g (Fig. 2b, c). The contents of α-carotene, β-carotene and phytoene increased rapidly at 200 DAF and 215 DAF, with values of 53.42, 27.90, and 10.57 μg/g, respectively, at 215 DAF. In addition, β-cryptoxanthin-laurate, rubixanthin-laurate β-cryptoxanthin, γ-carotene and others, were also increased at 215 DAF (Additional file 2: Data 5). Microsynteny analysis of carotenoid biosynthesis pathway genes showed that *MtSSUIIs* were triplicated and *MtCCD4s* were duplicated in the T genome (Figs. 1c and 3b). According to the RNA-seq data, the key genes of carotenoid biosynthesis, including *MtGGPPS1*, *MtSSUIIs*, *MtPSY2s*, *MtLCYBs*, *MtLCYEs*, *MtZDSs*, *Mtβ-OH* and *Mtε-OH*, were all highly expressed across all the fruit developmental stages (Fig. 3a, c). At 200 DAF and 215 DAF, hyperaccumulation of α-carotene, β-carotene and phytoene coincided with a decrease in *CCD4* expression.

The triplication of *MtSSUII* may explain the enrichment of carotenoids in karat and other Fe’i banana fruits. The lutein contents of these fruits were high throughout the fruit development process. *SSUII* enhances the accumulation of carotenoids by interacting with *GGPPS1* and *PSY*, promoting their enzymatic activity [31, 32]. In addition to *MtSSUIIs* enhancing the hyperaccumulation of carotenoids, *MtCCD4*, a key gene that regulates various branches of carotenoid biosynthesis, regulates the accumulation of α-carotene and β-carotene during ripening and is downregulated at the end of ripening [11]. Downregulation of *CCD4* is fruit-specific and may be the key reason for the enrichment of only α-carotene and β-carotene in the fruit. According to coexpression network analysis, *MtCCD4* was coregulated with *MtETO1* and *MtJAZ1*. *MtJAZ1* is the key regulator of the JA signalling pathway and is induced by JA [33]. Multiple JA response element G-box and TGACG-box motifs were identified in the promoters of *MtCCD4a* and *MtCCD4b* (Additional file 1: Fig. S13), similar to *CCD4* in *Brassica napus*, indicating an extensive role of JA in the regulation of *CCD4* [34]. In *Osmanthus fragrans*, *OfCCD4* were also induced by JA treatment [35]. In the full-green (FG) stage, the decreased expression level of *CCD4* coincides with the increase in the JA content, but in the full-ripening (FR) stage, the decrease in the JA content also coincides with the downregulation of *MtCCD4s*, implying the complex regulation of *MtCCD4s* by JA. For α-carotene and β-carotene rapidly accumulation in FR stage, JA may repress the accumulation of α-carotene and β-carotene by activating the expression of *MtCCD4s* in fruit. Therefore, further research is needed to elucidate the mechanism governing the regulation of *MtCCD4s* by JA, which may be spatiotemporally dependent and dose dependent.

#### Flavonoids are enriched early during fruit development, which may be due to the expansion of *MtF3′5′Hs*

**Fig. 4:**
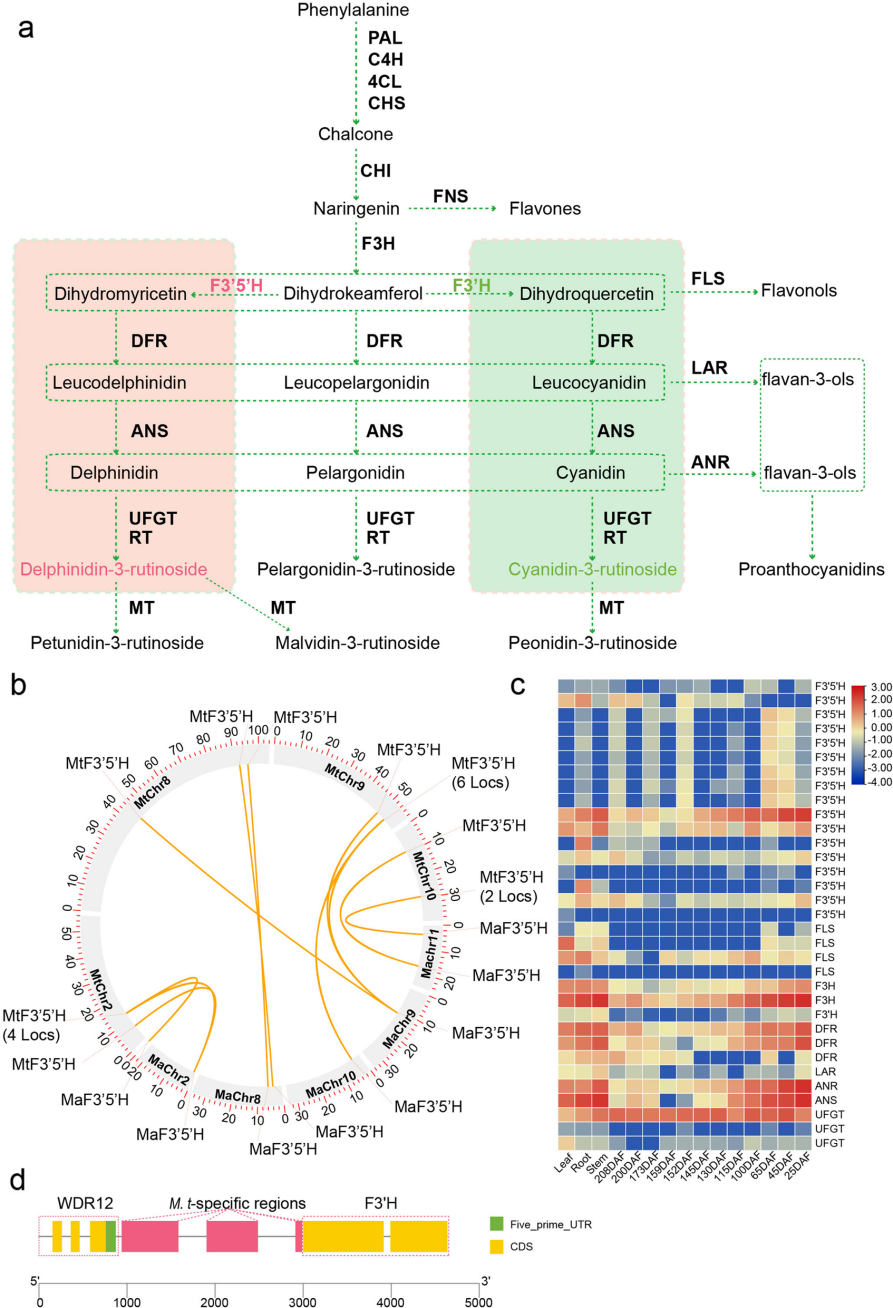
Schematic representation of the flavonoid biosynthesis pathway and duplication of key enzymes in karat. **a** Schematic representation of the flavonoid biosynthesis pathway. PAL, phenylalanine ammonia-lyase; C4H, cinnamate-4-hydroxylase; 4CL, 4-coumarate-CoA ligase; CHS, chalcone synthase; CHI, chalcone isomerase; F3H, flavanone 3-hydroxylase; F3′H, flavonoid 3′-hydroxylase; F3′5′H, flavonoid 3′,5′-hydroxylase; DFR, dihydroflavonol 4-reductase; ANS, leucoanthocyanidin dioxygenase; ANR, anthocyanidin reductase; LAR, leucoanthocyanidin reductase; FNS, flavone synthase; FLS, flavonol synthase; UFGT, anthocyanidin 3-O-glucosyltransferase; RT, UDP-rhamnose; and MT, anthocyanin O-methyltransferase. **b** Distribution of duplicated F3′5′Hs in the T and A genomes. **c** Heatmap of flavonoid biosynthesis pathway genes in different tissues. **d** Characteristic of the 5′ UTR upstream of F3′H

The T genome has 17 *F3′5 ′H* loci, while there are eight, eight and five loci in the A, B and S genomes respectively. Microsynteny analysis showed that the flavonoid biosynthesis gene *MtF3′5 ′H* was tandemly duplicated on both chromosomes 2 and 10, resulting in nine more loci than were present in the A genome (Fig. 4b). In particular, *F3′5′H* on chromosome 9, which is a single locus in the A, B and S genomes, is duplicated into eight loci in the T genome. Moreover, seven of the eight loci distributed on chromosome 9 of the T genome showed highly similar expression patterns in karat. *MtF3′H*, which competes with *MtF3′5′Hs* for substrates, was largely decreased in karat. Microsynteny analysis also showed that there are three *M. troglodytarum-*specific regions in the upstream sequences of *MtF3′H*. No similar sequences were identified by BLAST in A, B, S or other genomes. The specific regions may contribute to the low expression level of *MtF3’H* in karat. According to the quantification of flavonoids in pink stem sap, the delphinidin-3-rutinoside chloride content was enriched. Moreover, multiple flavonoids in the pulp were found to be enriched, including 4′-hydroxy-5,7-dimethoxyflavanone, epicatechin, myricetin-3-O-rutinoside, and delphinidin-3-O-rutinoside. In particular, only 4′-hydroxy-5,7-dimethoxyflavanone was enriched at 215 DAF, while epicatechin, myricetin-3-O-rutinoside and delphinidin-3-O-rutinoside degraded largely at the end of the ripening process, coinciding with the fading of pink sap in the fruit (Fig. 2a, c–e). The duplication of *MtF3′5′H* and suppression of *MtF3′H* led to the enrichment of delphinidin-3-O-rutinoside, which differs from other types of bananas. The heatmap shows that the key genes involved in the synthesis of flavonoids were downregulated at the end of the ripening process, except for *MtUFGTs*, which were highly expressed throughout the ripening process (Fig. 4a, c).

#### Riboflavin is enriched in karat pulp

**Fig. 5:**
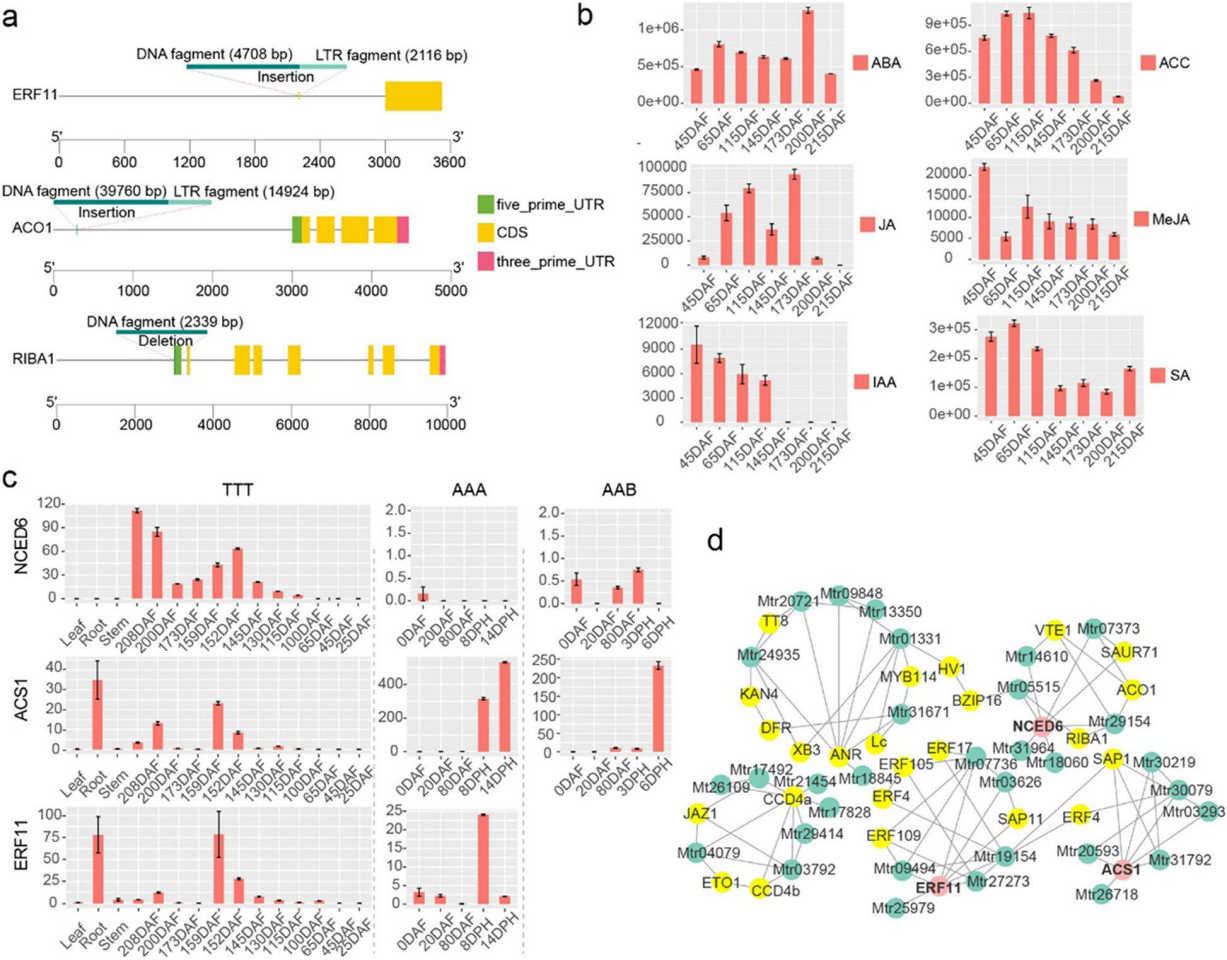
Modulation of fruit ripening by ABA and ethylene. **a** Characteristics of the ERF11, ACO1 and RIBA1 gene sequences. **b** Relative quantitation of plant hormones involved in fruit ripening. **c** Expression patterns of NCED6, ERF11 and ACS1. **d** Coexpression network of genes involved in the modulation of fruit ripening. DAF, days after flowering; DPH, days post-harvest

According to the widely targeted metabolome analysis, riboflavin (B2), pantothenic acid (B5) and pyridoxine (B6) were enriched in karat pulp (Fig. 2c). In particular, riboflavin (B2) was enriched, especially in Fe’i banana fruit. Transcriptome analysis also showed that the riboflavin de novo synthesis genes *MtRIBA1* and *MtFMNse* showed higher expression levels in karat fruit pulp than in BXJ (BaXi Jiao, *Musa acuminata* L. AAA group cv. Cavendish) fruit pulp. Moreover, microsynteny analysis showed that there was a 2399-bp deletion in the 5′ UTR of *MtRIBA1* (Fig. 5a). As a consequence, MtRIBA1 was highly expressed throughout all developmental stages, and the increased expression of *MtFMNSE* across the ripening process, may be the reason for the enrichment of riboflavin.

### Non-climacteric behaviour of karat fruit

Fruit ripening is distinctively different between climacteric and non-climacteric fruit. ABA and ethylene play key roles in the ripening of climacteric fruit, while non-climacteric ripening is linked to only ABA [36]. Banana is usually a climacteric fruit, while karat shows non-climacteric behaviour with a long bunch-filling time (215 days) and shelf life (harvested full yellow fruits can be stored for approximately 8 days under ambient conditions). By integrating metabolomic and comparative transcriptomic analyses, we found that the non-climacteric behaviour of karat is due to the transformation of ethylene-induced ripening into ABA-induced ripening.

Using a widely targeted metabolome, we quantified the plant hormones involved in the regulation of fruit development, including ABA, 1-aminocyclopropane-1-carboxylic acid (ACC), jasmonic acid (JA), indoleacetic acid (IAA), and salicylic acid (SA). ACC increased at 65 DAF and decreased at 145 DAF; ABA increased at 65 DAF and decreased at 215 DAF; JA increased at 65 DAF and decreased at 200 DAF; IAA decreased at 173 DAF; and SA decreased at 145 DAF but increased at 215 DAF (Fig. 5b). Karat fruit ripened at 215 DAF and was harvested at that time in June. At 65 DAF, homologues of *MaNAC1*, *MaBMY*, *MaAMY3*, *MaBMY3* and *MaBGAL6*, were upregulated with the onset of fruit ripening, and the ABA and JA contents increased as the fruits reached the mature-green or full-green (FG) stages. ABA may play key roles in both FG and full-ripening (FR) stage. While ethylene, IAA, JA and SA may involve in fruit ripening during the FG stage. Moreover, there was another ABA peak at 200 DAF, when the fruit reached the FR stage, coinciding with fruit softening and the rapid accumulation of α-carotene and β-carotene (Fig. 2a and Additional file 2: Data 6-8). Though ABA, ethylene, JA, IAA and SA were all reported to activate the biosynthesis of carotenoids in many plants [13, 35, 37, 38], ABA may be one of the key factors regulating the accumulation of α-carotene and β-carotene in FR stage.

Autocatalytic ethylene synthesis was disrupted in karat, but ABA synthesis was enhanced. *MaERF11* is a key ethylene-related gene that negatively regulates ethylene biosynthesis by suppressing *MaACS1* and *MaACO1* in banana [39]. *MaACS1* and *MaACO1* are the key genes that regulate ethylene biosynthesis. Homologues of *MaERF11*, MaACS1 and MaACO1 presented expression patterns that differed from those in FJ (Fen Jiao, *Musa* ABB PisangAwak) and BXJ (Fig. 5c). Both *MaERF11* and *MtERF11* are repressor with ERA repression motif ‘DLNNPP’. Microsynteny analysis showed that there was a DNA fragment of 4,708 bp and an LTR-like fragment of 2116 bp inserted 780 bp upstream of *MtERF11* and that there was a DNA fragment of 39,766 bp and an LTR-like fragment of 14,924 bp inserted 2720 bp upstream of *MtACO1* (Fig. 5a). Due to sequence variations, unlike that of *MaERF11* in BXJ, the expression of *MtERF11* was not suppressed during ripening as that of *MaERF11* in BXJ. The expression of *MtERF11* showed a similar expression pattern as that of *MtACS1* during ripening, which is unlike the sharp upregulation of *ACS1* in FJ and BXJ. This result indicated that *MtERF11* suppressed the expression of *MtACS1* throughout the fruit development process. The promoter sequence of *MtACS1* harbour GCC-boxes, while that of *MtACO1* lacks GCC-boxes, which may explain the upregulation of *MtACO1* during ripening (Additional file 1: Fig. S14). Analysis of the cloned promoter sequences of *MtACO1* also validated the missing of GCC-boxes. As a downstream gene of the carotenoid synthesis pathway and a gene that catalyses the first step of ABA biosynthesis, *MtNCED6* may play key roles in the regulation of ABA synthesis in karat fruit, as this gene is specifically expressed during ripening, unlike in FJ and BXJ [40]. Moreover, unlike in FJ and BXJ, the metabolism-related gene *MtCYP707A1* was not upregulated during ripening. It may be the key gene involved in the decrease in ABA content in BXJ during ripening. *MtCYP707A2* was upregulated at 208 DAF, which may explain the decrease in ABA levels (Additional file 1: Fig. S15-17). ABA-stress-ripening (ASR) transcription factors play key roles in sucrose- and ABA-induced fruit ripening and softening via crosstalk between ABA and sucrose [41]. In BXJ, *MaASR1* and *MaASR2* increased before ripening but sharply decreased, while they were highly expressed during karat fruit ripening. *MtASR1* expression was maintained at a high level throughout the ripening period, indicating that this gene may play a role in karat fruit ripening and softening.

**Fig. 6:**
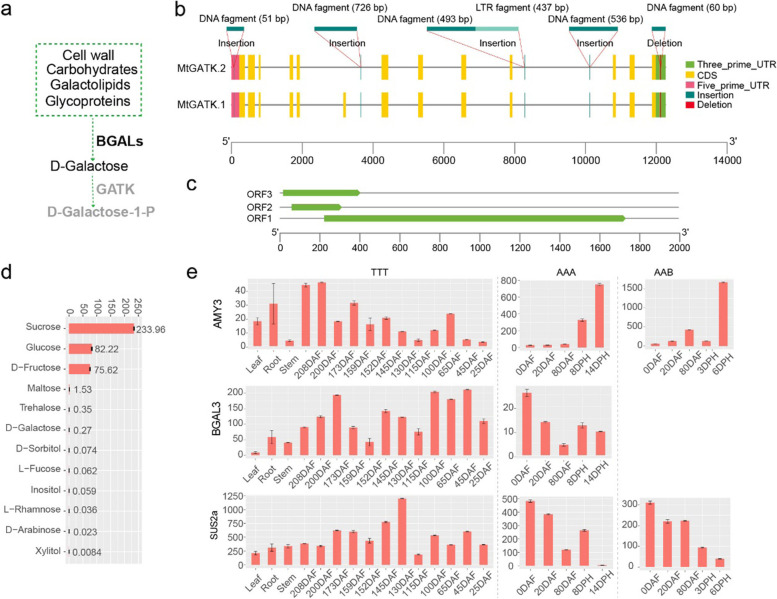
Accumulation of sugars in karat. **a** Schematic representation of D-galactose biosynthesis in karat. GATK, galactokinase; BGAL, β-galactosidase. **b** Alternative splicing and characteristics of GATK gene sequences. **c** Three ORFs in the mRNA of GATKs. **d** Quantification of sugars in the pulp of ripe fruit. **f** Expression patterns AMY3 and BGAL3. DAF, days after flowering; DPH, days post-harvest

As starch was degraded and converted into soluble sugars during ripening, karat had lower glucose and fructose contents but higher sucrose and free galactose contents than BXJ, due to non-climacteric behaviour. Quantification of sugars in the pulp of ripening karat showed that sucrose was highly enriched, coinciding with the degradation of starch. In the pulp of dry ripened fruit, the sucrose content reached 233.96 mg/g and was the predominant sugar (Fig. 6d and Additional file 2: Data 9). Given that the pulp of the ripening fruit is 67% water [4], karat pulp has approximately 27.13 and 24.95 mg/g glucose and fructose, respectively, which is less than the approximately 60 mg/g glucose and fructose found in BXJ [42]. During ripening, the starch-degradation-related genes *MtBMYs* and *MtAMYs* were upregulated, coinciding with the accumulation of sucrose, fructose and glucose. However, in contrast to that of their homologues in BXJ and FJ, the expression of these genes did not sharply increase in karat (Fig. 6e and Additional file 1: Fig. S18). Sucrose synthase genes were highly expressed in karat during ripening, which differed from the decrease in BXJ and FJ. Moreover, the level of free galactose in the pulp at ripening was 0.27 mg/g. The galactose synthesis-related genes *Mtα-GALs* and *Mtβ-GALs* had higher expression levels than those in BXJ and FJ, which may be due to the non-climacteric behaviour. *GALK* is involved in galactose metabolism [43]. *Compared to MaGALK, MtGALK* is a 5′ prime untranslated region (UTR) premature start codon-gain variant that has two premature ORFs in the 5′ UTR (Fig. 6a–c). Premature ORFs may suppress the translational activity of transcripts [44]. *MtGALK* also has an alternative transcript in which the sixth exon is missing, which may be due to a DNA sequence insertion in the sixth intron (Fig. 6c). The cloned *MtGALK* cDNA sequences validated the variation of in 5′ UTR and alternative splice. Moreover, the karat shoot buds presented better growth vigour than BXJ under exogenous application free galactose (Additional file 1: Fig. S19). Therefore, the free galactose content in the pulp was higher than that in the pulp of other banana fruits and may be due to sequence variations in the 5’ UTR and sixth intron of *MtGALK*. Non-climacteric ripening behaviour may be another reason for the accumulation of free galactose; similar to the fruit of non-climacteric plum cultivars, which shows increased expression levels of α-GALs and β-GALs and increased accumulation of free galactose [45].

Using weighted gene coexpression network analysis (WGCNA), we constructed a weighted coexpression network. To identify candidate genes involved in fruit development and ripening, flavonoid and carotenoid biosynthesis, we selected *MtXB3*, *MtNAC1*, *MtCCD4s*, *MtERFs*, *MtNCED6*, and *MtDFR* to extract subnetwork from coexpression network (Fig. 5d and Additional file 2: Data 10). *MtXB3*, a homologue of *MaXB3* that negatively regulates fruit ripening, was downregulated at 130 DAF. *MtXB3* was coexpressed with *MtTT8*, *MtLc*, *MtMYB114*, *MtDFR*, and *MtANR*, which may play key roles in flavonoid biosynthesis. *MtERF11* and *MtACS1* were coexpressed with *MtSAP1*, *MtSAP4*, *MtERF4*, *MtERF105*, *MtERF107* and *MtERF109*. *MtNCED6* was coregulated with *MtRIBA1*, *MtACO1*, *MtSAUR1*, *MtCCD4a* and *MtCCD4b*. And *MtCCD4a* and *MtCCD4b* negatively regulate the accumulation of α-carotene and β-carotene and were coregulated with *MtJAZ1* and *MtETO1*.

## Discussion

Karat is an edible parthenocarpic cultivar of the Australimusa section known as Fe’i banana. Different from the known karyotype of Pisang tongkat langit, in which TT and Asupina were combined with ATT, in karat, these genomes were combined with TTT. In this study, we constructed a high-quality T genome with a size of 603 Mb, representing 92% of the estimated genome size of 655 Mb. Transposable element (TE) contents comprise 59.62% ,45.16%, 49.55% and 56.69% of the T, A, B and S genomes, respectively [16–18]. The T genome was larger than the A, B and S genomes (523, 521 and 587 Mb, respectively), which may be due to the presence of more TE contents. This is especially true for LTR/Copia (36.41%) and LTR/Gypsy (15.07%), which account for 51.48% of the T genome. Through chromosome rearrangement analysis of the T genome, we found that ancestral chromosomes 8 and 9 fused into modern chromosome 9. Phylogenetic analysis based on orthologous genes shows that the divergence time between *M. troglodytarum* and the ancestor of *M. acuminata* and *M. schizocarpa* was about 20.8 MYA, indicating a much earlier divergence time between Callimusa and Musa than previous reported 37.9-50.7 MYA [46–48]. This may be because of utilizing more informative characters to construct phylogentetic trees in genome-wide studies. After divergence from the ancestor of *M. acuminata* and *M. schizocarpa*, the *M. troglodytarum* genome experienced multiple chromosome rearrangements and sequence variations, which resulted in Fe’i banana fruit having many specific features, such as a red or purple sap rich in delphinidin-3-rutinoside chloride, a deep yellow-orange-coloured flesh that is rich in β-carotene and riboflavin, high contents of galactose, and a non-climacteric ripening pattern. By integrating metabolome, comparative transcriptome and microsynteny analyses, we revealed the genomic basis of these features, which provides insights into the improvement of nutritional and bioactive qualities, the prolonging of fruit shelf-life and reduction in postharvest crop losses.

Some fruit, such as those of melon, pepper, plum, and pear, show both climacteric and non-climacteric behaviour. In the non-climacteric varieties, endogenous ethylene synthesis is suppressed [49–53]. Banana is usually a climacteric fruit, while karat shows non-climacteric behaviour with the transformation of ethylene-induced ripening into ABA-induced ripening. The triplication of *MtSSUII* resulted in the enhancement of ABA biosynthesis, and the suppression of ethylene biosynthesis by a mutation in *MtERF11* may be the primary reason for the non-climacteric behaviour of karat. In banana, a complex network regulating fruit ripening has been reported, and a dual-loop circuit, involving NAC and MADS transcription factors and ethylene signalling, was shown to control banana fruit ripening [54]. *MaMADS1*, *MaMADS2* and *MaMADS36* are also necessary for banana fruit ripening [55–57]. However, the expression patterns of *MtMADS1*, *MtMDAS2a* and *MtMADS2b*, which are homologues *MaMADS1* and *MaMADS2*, were different from those in BXJ; thus, these genes may function in an ethylene-dependent mode. A multilayered regulatory cascade comprising *MaNAC1*, *MaNAC2*, *MaXB3*, *MaERF11*, *MaACS1* and *MaACO1* is involved in ripening, of which *MaERF11* functions as a repressor of ethylene biosynthesis by repressing *MaACS1* and *MaACO1* [39, 58, 59]. *MaERF11* was repressed by *MaNAC2* and *MaNAC1*; both genes were repressed by *MaXB3*. During ripening, the repression of *MaACS1* was removed with the downregulation of *MaXB3*. However, in karat, the regulatory cascade was disrupted by the insertion of LTRs, and *MtERF11* repressed the expression of *MtACS1* throughout the fruit development process. Additionally, the comparative transcriptome analysis indicated that the regulatory network comprising *NAC1, NAC2*, *XB3*, *ERF11*, *ACS1* and *ACO1* was conserved in Fj, BXJ and karat. Moreover, ABA-related transactors such as *ABI5*, *ASR1*, and *ASR2* were also involved in the regulation of fruit ripening in Fj, BXJ and karat. These data will provide important resources for further research on the fruit ripening process of banana.

## Conclusions

In this study, we de novo sequenced the genome of *Musa troglodytarum* L. for the first time. Genome microsynteny analysis showed that the duplication of *MtSSUIIs* due to chromosome rearrangement may lead to the accumulation of carotenoids and ABA in the fruit. And the expression of duplicated *MtCCD4s* is repressed during ripening, leading to the accumulation of α-carotene, β-carotene and phytoene. The duplication of *MtF3′5′H* and suppression of *MtF3′H* led to the enrichment of delphinidin-3-O-rutinoside, which is different from that in the other types of bananas. The free galactose content in the pulp was higher than that in the pulp of other banana fruit and may be due to the sequence variations in the 5’ UTR and sixth intron of *MtGALK*. Due to an LTR fragment insertion upstream of *MtERF11*, karat cannot produce large amounts of ethylene but can produce ABA during ripening, resulting in non-climacteric behaviour and prolonging bunch filling time. In conclusion, integrating metabolome, comparative transcriptome and microsynteny analysis, we revealed the genomic basis of non-climacteric behaviour and enrichment of carotenoids, riboflavin, flavonoids, free galactose of karat (*Musa troglodytarum* L.). These data provide insights into the improvement of the nutritional and bioactive qualities, prolongation of shelf-life and reducing postharvest crop loss.

## Methods

### Sampling, sequencing and assembly

Karat plants growing in a greenhouse of the National Gene Bank of Tropical Crops in Danzhou, Hainan, China, were sampled for genome sequencing. This *Musa* germplasm was collected from Kosrae of The Federated States of Micronesia during a plant resources investigation and cooperation. The genomic DNA of leaves was extracted for genomic library construction. The DNA high-molecular-weight genomic DNA extraction was performed using an SDS-based method [60]. For Illumina sequencing, libraries with 350 bp insertions were constructed. For PacBio sequencing, libraries with 20,000 bp insertions were constructed and sequenced on the PacBio RS II system using P6-C4 chemistry. For Nanopore single-molecule sequencing, libraries with high-molecular-weight genomic DNA were constructed on PromethION. In total, 42,304,446,276 bp reads were produced by Nanopore single-molecule sequencing, 42,215,455,800 bp Illumina short reads were produced, and 6,961,206,933 bp PacBio reads were produced.

Hi-C libraries were created from young leaves of *M. troglodytarum* at BioMarker Technologies Company as described previously [61]. Briefly, the leaves and formaldehyde were mixed together and then lysed, and then the cross-linked DNA was digested with *DpnII* overnight. Sticky ends were biotinylated and proximity-ligated to form chimaeric junctions that were enriched and then physically sheared to a size of 500–700 bp. Chimaeric fragments representing the original cross-linked long-distance physical interactions were then processed into paired-end sequencing libraries, and 110 Gb of paired-end reads were produced on the Illumina HiSeq X Ten platform.

For RNA-seq, total RNA was extracted from leaves, roots, pseudostems and fruits using TRIzol reagent according to the manufacturer’s instructions. After removing genomic DNA using *DNase I* (Takara), mRNAs were obtained using oligo (dT) beads and subsequently broken into short fragments, followed by cDNA synthesis. Paired-end sequencing was conducted on a HiSeq X Ten platform (Illumina, CA, USA).

### Genome assembly and annotation

Using GenomeScope 2.0 [62], a program that employs a polyploid-aware mixture model to assess heterozygous and polyploid genomes, we estimated the genome size using jellyfish-produced K-mer counts. Nanopore long reads were imported for assembly by NextDenovo v.2.3.0 (https://github.com/Nextomics/NextDenovo), with the errors first corrected by the NextCorrect module and then assembled into 918,212,466 bp contigs by the NextGraph module. Utilizing Illumina short reads and PacBio reads, NextPolish (https://github.com/Nextomics/NextPolish) was subsequently used to polish the preliminary assembly with the default parameters. Then, the allelic haplotigs were eliminated by Purge_Haplotigs (V1.1.1) [63]. The final assembly was then assembled into a scaffold based on proximity-guided assembly by ALLHIC [64].

RepeatModeler v1.0.11 with default parameters was used to identify TEs de novo [65]; this program employs two different software programs: RECON (v1.08) and RepeatScout (v1.0.5). For consensus building and classification steps, the consensus TE libraries generated above were imported into MAKER2 for further repeat annotations by employing RepeatMasker (v4.05). TEclass (v2.1.3) was subsequently used to classify unknown TEs using a support vector machine (SVM) method. Tandem Repeat Finder (v4.07) was then used to identify repeats within the genomes with the following parameters: ‘1 1 2 80 5 200 2,000 –d –h’.

To detect LTRs, the LTR_retriever pipeline with default parameters was used to integrate and remove false positives from the initial predictions of LTR FINDER [27, 66]. BUSCO v5.22 was used to evaluate genome completeness using the viridiplantae_odb10 dataset, which includes 425 single-copy and conserved protein-coding genes [67]. The LTR insertion time was estimated by the transcripts implemented in the LTR package using the formula T = K/2μ, with μ representing the neutral mutation rate and set to 1.38 × 10^−8^.

The repeats and protein-coding genes in the genome were annotated by MAKER2 (v3.01.02) [23]. The MAKER2 pipeline was processed twice to obtain high-quality gene annotations. The RNA-seq reads of the leaves, stems, roots and fruits were imported to Trinity to generate genome-guided and de novo assemblies, with the default parameters used. Then, the assemblies were imported into the PASA pipeline (v2.3.3) to construct a comprehensive transcript database. The comprehensive transcripts were used to train the predictors embedded in the MAKER2 pipeline, including AUGUSTUS (v3.3.1), GENEMARK (v3.5.2) and SNAP (version 2006-07-28). After filtering out proteins produced by MAKER2 that had low AED values, the ab initio predictors AUGUSTUS, GENEMARK and SNAP were trained again. Then, utilizing high-confidence and high-sensitivity transcripts produced by StringTie (v2.1.1) [68] as inputs, the MAKER2 pipeline was run again. Using eggNOG-mapper (v2) [24] and its default database, genes were assigned functional annotations.

### Genome structure and evolution

The genome data of *M. acuminata* (GCA_000313855.2) [69], *M. balbisiana* (GCA_004837865.1) [70] and *M. itinerans* (GCA_001649415.1) [71] was downloaded from NCBI. The genome data of *M. schizocarpa* was downloaded at http://www.genoscope.cns.fr/plants [72]. The RNA-seq data of BXJ and FJ were downloaded from BioProject accession number PRJNA394594. The short reads genome sequencing data included in SRR15675960 for tongkat and SRR16526594 for karat were used to estimate genome size.

Orthologues were identified by OrthoFinder (v2.2.7) [28] with default parameters, using the longest transcripts of protein-coding genes from *M. troglodytarum*, *M. acuminata*, *M. balbisiana*, *M. schizocarpa* and M. itinerans. According to the orthologue set mentioned above and the results from MCScanX (v0.8) [73], putative protogenes (pPGs) were predicted. Then, the pPGs ordered according to the gene location in each species were integrated into GRIMM format and imported into MGRA2 [30] for ancestral genome reconstruction, in which heuristic higher breakpoint reuse was used. Then, the exhaustive set of ordered protogenes (oPGs) was imported for collinear genes reported by MCScanX [73]. The rooted tree generated by OrthoFinder was used for the construction of ultrametric trees of *M. troglodytarum*, *M. acuminata*, *M. balbisiana* and *M. schizocarpa* by r8s (v1.81) [74] with default parameters. Using Café (v4.2.1) [29] with default parmameters, we subsequently estimated the gene family expansion and contraction events among *M. troglodytarum*, *M. acuminata*, *M. balbisiana* and *M. schizocarpa*. Using R packages clusterProfiler [75] with parameters “pvalueCutoff = 0.05, pAdjustMethod = ‘BH’”, we performed GO enrichment of *M. troglodytarum* specific genes and rapidly evolving families.

### Metabolomic analyses

Carotenoid, flavonoid, monosaccharide and disaccharide contents were analysed via targeted metabolomics. Karat pulp at 215 DAF was used for monosaccharide and disaccharide analysis. The pink sap of karat was used for flavonoid and carotenoid analysis. Karat pulp at 25, 45, 65, 115, 145, 173, 200 and 215 DAF was used for carotenoid analysis. The carotenoid, flavonoid, monosaccharide and disaccharide contents were analysed as described in previous studies [76–78]. The sample extracts were analysed using a UPLC-ESI-MS/MS system (ExionLC™ AD https://sciex.com.cn/; MS, Applied Biosystems 6500 Triple Quadrupole, https://sciex.com.cn/). Linear ion trap (LIT) and triple quadrupole (QQQ) scans were acquired on a QQQ-linear ion trap mass spectrometer (QTRAP), QTRAP® 6500+ LC–MS/MS system equipped with an ESI turbo ion-spray interface operating in positive ion mode and controlled by Analyst v1.6.3 software (AB-SCIEX).

For widely targeted metabolomics analyses, karat pulp at 25, 45, 65, 115, 145, 173, 200 and 215 DAF was used. The metabolomics analyses were performed as described in a previous study [79]. Briefly, the sample extracts were analysed using a UPLC–ESI–MS/MS system (Shimadzu Nexera X2, https://www.shimadzu.com.cn/; MS, Applied Biosystems 4500 QTRAP, https://www.thermofisher.cn/cn/zh/home/brands/applied-biosystems.html). LIT and QQQ scans were acquired on a triple quadrupole-linear ion trap mass spectrometer (QTRAP), AB4500 QTRAP UPLC/MS-MS system was equipped with an ESI turbo ion-spray interface operating in positive and negative ion mode and controlled by Analyst 1.6.3 software (AB-SCIEX).

## Supplementary Information

**Additional file 1: Figure S1**. Fluorescent staining of karat chromosomes. The root tip of *Musa troglodytarum L.* karat plants staining with DPAI and screened under ultraviolet and fluorescence microscopy. **Figure S2**. GenomeScope profile of karat and tongkat. **Figure S3**. Bimodal histogram for Purge Haplotigs processing. The cutoff values for low, mid, and high points were 6,42 and 105, respectively. **Figure S4**. Hi-C mapping of chromosomes of the T genome. **Figure S5**. Heatmap of density of Nanica LINE. **Figure S6**. Analysis of LTR insertion times of the A, B, S and T genomes. Ma, *Musa acuminata*; Mb, *Musa balbisiana*; Ms, *Musa schizocarpa*; and Mt, *Musa troglodytarum* L. **Figure S7**. Distribution of SNP and indel sites in karat and tongkat. Indel sites in tongkat (A) and karat (B) and SNP sites in tongkat (C) and karat (D). **Figure S8**. GO enrichment analysis of T genome specific genes. **Figure S9**. GO enrichment analysis rapidly evolving gene families in T genome. **Figure S10**. Dot plot of syntenic bocks between *M. troglodytarum* and *M. acuminata*. **Figure S11**. Synteny map of chromosome 8 and 9 among *M. troglodytarum*, *M. acuminata* and *M. balbisiana*. **Figure S12**. Clusters of metabolites in karat fruit pulp. DAF, days after flowering. **Figure S13**. The distribution of JA response element G-box and TGACG-box motifs in the promoters of *MtCCD4s*. **Figure S14**. The distribution of GCC-boxes in the promoters of *MtACO1* and *MtACS1*. **Figure S15**. Expression patterns of genes involved in karat fruit ripening. Gene expression was normalized to FPKM (fragments per kilobase of transcript per million read pairs). DAF, days after flowering. **Figure S16**. Expression patterns of genes involved in FJ fruit ripening. Gene expression was normalized to FPKM (fragments per kilobase of transcript per million read pairs). FJ (Fen jiao), dwarf banana. DAF, days after flowering. DPH, days post-harvest. **Figure S17**. Expression patterns of genes involved in BXJ fruit ripening. Gene expression was normalized to FPKM (fragments per kilobase of transcript per million read pairs). BXJ (BaXi Jiao), Cavendish banana. **Figure S18**. Expression patterns of genes involved in starch degradation and galactose accumulation. The gene IDs of M. troglodytarum, M. acuminata and M. balbisiana start with ‘Mt’, ‘LOC’ and ‘THU’, respectively. Gene expression was normalized to FPKM (fragments per kilobase of transcript per million read pairs). **Figure S19**. The effect of exogenous application free galactose on shoot buds of karat and BXJ. The shoot buds of karat (a) and BXJ (b) grown 20 days under a controlled environment (10 h light, 35% relative humidity and 25 °C). The karat (c)and BXJ (d) shoot buds (2-3 cm) were transferred into half-strength MS supplemented with Gal (10 mM, B2; and 100 mM, B1) and sucrose (10 mM, Z1), respectively. **Table S1**. Summary of GenomeScope profile on Tongkat with a k-mer of 19. **Table S2**. Summary of GenomeScope profile on karat with a k-mer of 19. **Table S3**. summary of short reads of genome sequencing. **Table S4**. summary of Pacbio reads of genome sequencing. **Table S5**. Summary of Nanopore reads of genome sequencing. **Table S6**. Summary of BUSCO analysis of contigs before Purge Haplotigs processing (C:98.4%). **Table S7**. Summary contigs after Purge Haplotigs processing and correction of chimeric contigs using ALLHIC_correcter. **Table S8**. Summary of BUSCO analysis of genome (C:97.7%). **Table S9**. Summary of protein-coding genes of M. *troglodytarum.* **Table S10**. Summary of BUSCO analysis of predicted gene (C:92.5%). **Table S11**. Summary of Repeat content of genome. **Table S12.** Summary of SNP and Indel sites of Karat and Tongkat.

**Additional file 2: Data 1**. List of M. *troglodytarum* specific genes. Genes were annotated by eggNOG-mapper. **Data 2**. List of genes of rapidly evolving families in M. *troglodytarum.* Genes were annotated by eggNOG-mapper. **Data 3**. Metabolites of karat pulp identified by widely targeted metabolomics analysis. **Data 4**. Clustered metabolites of karat pulp identified by widely targeted metabolomics analysis. **Data 5**. Carotenoid contents of pulp at different developmental stages. **Data 6**. Expression levels of genes involved in fruit ripening in karat. **Data 7**. Expression levels of genes involved in fruit ripening in BXJ. **Data 8**. Expression levels of genes involved in fruit ripening in FJ. **Data 9**. Sugar contents in the pulp of ripening karat fruit. **Data 10**. Expression levels of nodes of the ripening-related coexpression network.

## Data Availability

The *Musa troglodytarum L*. genome sequences and raw sequence data from RNA-seq with accessions SRX12729179-SRX1272927 and genome assembly with assession ASM2354706v1 have been deposited under BioProject accession number PRJNA772907 [80]. The GFF format Gene and TE annotation file of the *Musa troglodytarum L*. genome was available in figshare [81]. The cloned promoter sequences of *MtACS1*, *MtACO1* and cDNA sequences of *MtGALK* and *MtSSUIIs* were deposited in figshare [82].
