# Supplementary material for: The Musa troglodytarum L. genome provides insights into the mechanism of non-climacteric behaviour and enrichment of carotenoids
Source: BMC Biol. 2022 Aug 24;20:186. doi: 10.1186/s12915-022-01391-3 (PMC9400310; doi:10.1186/s12915-022-01391-3)
Supplement: Supplementary file 1 — Additional file 1: Figure S1. Fluorescent staining of karat chromosomes. The root tip of Musa troglodytarum L. karat plants staining with DPAI and screened under ultraviolet and fluorescence microscopy. Figure S2. GenomeScope profile of karat and tongkat. Figure S3. Bimodal histogram for Purge Haplotigs processing. The cutoff values for low, mid, and high points were 6,42 and 105, respectively. Figure S4. Hi-C mapping of chromosomes of the T genome. Figure S5. Heatmap of density of Nanica LINE. Figure S6. Analysis of LTR insertion times of the A, B, S and T genomes. Ma, Musa acuminata; Mb, Musa balbisiana; Ms, Musa schizocarpa; and Mt, Musa troglodytarum L. Figure S7. Distribution of SNP and indel sites in karat and tongkat. Indel sites in tongkat (A) and karat (B) and SNP sites in tongkat (C) and karat (D). Figure S8. GO enrichment analysis of T genome specific genes. Figure S9. GO enrichment analysis rapidly evolving gene families in T genome. Figure S10. Dot plot of syntenic bocks between M. troglodytarum and M. acuminata. Figure S11. Synteny map of chromosome 8 and 9 among M. troglodytarum, M. acuminata and M. balbisiana. Figure S12. Clusters of metabolites in karat fruit pulp. DAF, days after flowering. Figure S13. The distribution of JA response element G-box and TGACG-box motifs in the promoters of MtCCD4s. Figure S14. The distribution of GCC-boxes in the promoters of MtACO1 and MtACS1. Figure S15. Expression patterns of genes involved in karat fruit ripening. Gene expression was normalized to FPKM (fragments per kilobase of transcript per million read pairs). DAF, days after flowering. Figure S16. Expression patterns of genes involved in FJ fruit ripening. Gene expression was normalized to FPKM (fragments per kilobase of transcript per million read pairs). FJ (Fen jiao), dwarf banana. DAF, days after flowering. DPH, days post-harvest. Figure S17. Expression patterns of genes involved in BXJ fruit ripening. Gene expression was normalized to FPKM (fragments pe [file 12915_2022_1391_MOESM1_ESM.docx]

**The *Musa*** ***troglodytarum* L. genome provides insights into the mechanism of non-climacteric behaviour and enrichment of carotenoids**

Zhiying Li^1,2,3,4, †^, Jiabin Wang^1,2,3,4, †^, Yunliu Fu^1,2,3,4^, Yonglin Jing^1,2,3,4^, Bilan huang^1,2,3,4^, Ying Chen^5^, Qinglong Wang^1^, , XiaoBing Wang^1,2,3,4^, Chunyang Meng^1,2,3,4^, Qingquan Yang^1,2,3,4^ & Li Xu^1,2,3,4, *^

†These authors contributed equally to this work.

*Corresponding author

Email: [xllzy@263.net](mailto:xllzy@263.net)

1 Institute of Tropical Crop Genetic Resources, Chinese Academy of Tropical Agricultural Sciences, Danzhou 571737, Hainan, China.

2 Ministry of Agriculture Key Laboratory of Crop Gene Resources and Germplasm Enhancement in Southern China, Danzhou, 571737, Hainan, China.

3 Hainan Province Key Laboratory of Tropical Crops Germplasm Resources Genetic Improvement and Innovation, Danzhou 571737, Hainan, China.

4 National Gene Bank of Tropical Crops, Danzhou, 571700, Hainan, China.

5 College of Horticulture and Landscape Architecture, Hainan University, Haikou, 570228, China.

Table S1 Summary of GenomeScope profile on Tongkat with a k-mer of 19.

| property | min | max |
| --- | --- | --- |
| Homozygous (aa) | 98.4728% | 98.7589% |
| Heterozygous (ab) | 1.24111% | 1.52722% |
| aab | 1.24111% | 1.40428% |
| abc | 0% | 0.122935% |
| Genome Haploid Length | 651,288,318 bp | 655,194,593 bp |
| Genome Repeat Length | 374,597,237 bp | 376,843,983 bp |
| Genome Unique Length | 276,691,081 bp | 278,350,609 bp |
| Model Fit | 46.0232% | 97.8743% |
| Read Error Rate | 0.217203% | 0.217203% |

Table S2 Summary of GenomeScope profile on karat with a k-mer of 19.

| property | min | max |
| --- | --- | --- |
| Homozygous (aa) | 98.7271% | 99.0177% |
| Heterozygous (ab) | 0.982281% | 1.27292% |
| Genome Haploid Length | 597,280,676 bp | 606,906,342 bp |
| Genome Repeat Length | 335,016,792 bp | 340,415,861 bp |
| Genome Unique Length | 262,263,885 bp | 266,490,482 bp |
| Model Fit | 48.3717% | 98.5423% |
| Read Error Rate | 0.523735% | 0.523735% |

Table S3 summary of short reads of genome sequencing.

| Library strategy | Hi-C | WGS |
| --- | --- | --- |
| Library layout | PAIRED | PAIRED |
| Platform | ILLUMINA | ILLUMINA |
| Instrument | HiSeq X Ten | HiSeq X Ten |
| Total number of spots | 367575880 | 140,718,186 |
| Total number of bases(bp) | 110,272,764,000 | 42,215,455,800 |
| GC percentage | 41.7% | 40.07% |
| Insert size peak(bp) | 269 | 269 |
| Read length(bp) | 150 | 150 |
| Depth | 182.6 | 69.9 |

Table S4 summary of Pacbio reads of genome sequencing.

| Library strategy | WGC |
| --- | --- |
| Library layout | SINGLE |
| Platform | PACBIO SMRT |
| Instrument | PacBio RS II |
| Total number of spots | 749,340 |
| Total number of bases(bp) | 6,961,206,933 |
| GC percentage | 40.69% |
| Average length(bp) | 9289.8 |
| Max length(bp) | 213,087 |
| Depth(x) | 11.5 |

Table S5 summary of Nanopore reads of genome sequencing.

| Library strategy | WGC |
| --- | --- |
| Library layout | SINGLE |
| Platform | OXFORD NANOPORE |
| Instrument | PromethION |
| Total number of spots | 1,946,752 |
| Total number of bases(bp) | 42,304,446,276 |
| GC percentage | 39.20% |
| Average length(bp) | 21,730.8 |
| Max length(bp) | 203,887 |
| Base calling | Guppy v3.2.2 |
| N50(bp) | 28,726 |
| Depth(x) | 70.0 |

Table S6 Summary of BUSCO analysis of contigs before Purge Haplotigs processing (C:98.4%).

| 418 | Complete BUSCOs (C) |
| --- | --- |
| 249 | Complete and single-copy BUSCOs (S) |
| 169 | Complete and duplicated BUSCOs (D) |
| 4 | Fragmented BUSCOs (F) |
| 3 | Missing BUSCOs (M) |
| 425 | Total BUSCO groups searched |

Table S7 summary contigs after Purge Haplotigs processing and correction of chimeric contigs using ALLHIC_correcter.

| contigs (>= 0 bp) | 1150 |
| --- | --- |
| contigs (>= 1000 bp) | 1150 |
| contigs (>= 5000 bp) | 712 |
| contigs (>= 10000 bp) | 661 |
| contigs (>= 25000 bp) | 596 |
| contigs (>= 50000 bp) | 553 |
| Largest contig | 43024999 |
| Total length | 603485406 |
| Total length (>= 0 bp) | 603485406 |
| Total length (>= 1000 bp) | 603485406 |
| Total length (>= 5000 bp) | 602813406 |
| Total length (>= 10000 bp) | 602483406 |
| Total length (>= 25000 bp) | 601517406 |
| Total length (>= 50000 bp) | 600056406 |
| N50 | 6002247 |
| N75 | 1817000 |
| L50 | 24 |
| L75 | 68 |
| GC (%) | 39.58 |

Table S8 Summary of BUSCO analysis of genome (C:97.7%).

| 415 | Complete BUSCOs (C) |
| --- | --- |
| 393 | Complete and single-copy BUSCOs (S) |
| 22 | Complete and duplicated BUSCOs (D) |
| 5 | Fragmented BUSCOs (F) |
| 5 | Missing BUSCOs (M) |
| 425 | Total BUSCO groups searched |

Table S9 summary of protein-coding genes of *M. troglodytarum*.

| Protein coding gene number | 37,577 |
| --- | --- |
| Total length of protein coding gene (bp) | 192,766,275 |
| Average length of protein coding gene (bp) | 5,129 |
| Total exon length (bp) | 64,862,008 |
| Average length of exon (bp) | 316 |
| Total intron length (bp) | 127,904,267 |
| Average length of intron (bp) | 818 |
| Genes with alternative splice transcripts | 13,357 |
| Genes with one more exon | 31,360 |

Table S10 Summary of BUSCO analysis of predicted gene (C:92.5%).

| 393 | Complete BUSCOs (C) |
| --- | --- |
| 203 | Complete and single-copy BUSCOs (S) |
| 190 | Complete and duplicated BUSCOs (D) |
| 24 | Fragmented BUSCOs (F) |
| 8 | Missing BUSCOs (M) |
| 425 | Total BUSCO groups searched |

Table S11 Summary of Repeat content of genome.

|  |  | Number | length | % of repeats | % of geneome |
| --- | --- | --- | --- | --- | --- |
|  | **Repeats total** | 201646 | 367133459 | 100 | 60.8338 |
| LTR Retrotransposon | Gypsy | 11839 | 90963345 | 24.77664 | 15.07257 |
|  | Copia | 22314 | 219749689 | 59.85553 | 36.4124 |
|  | unknown | 2628 | 10755024 | 2.929459 | 1.782101 |
| Retrotransposon | LINE | 4984 | 3343741 | 0.9107699 | 0.554056 |
|  | SINE | 1 | 59 | 1.607045e-05 | 9.776266e-06 |
| Unclassified retroelement | Unknown | 54756 | 32663669 | 8.896947 | 5.412351 |
| DNA | hAT-Ac | 529 | 301732 | 0.08218592 | 0.04999682 |
|  | hAT-Tag1 | 1573 | 2047178 | 0.5576114 | 0.3392162 |
|  | MuLE-MuDR | 502 | 304752 | 0.08300851 | 0.05049723 |
| **TE total** |  | 99126 | 359824437 | 98.00916 | 59.6227 |
|  | Simple_repeat | 102520 | 7309022 | 1.990835 | 1.211101 |

Table S12 Summary of SNP and Indel sites of Karat and Tongka

| Tongka INDEL sites | 459,137 |
| --- | --- |
| karat INDEL sites | 516,884 |
| Tongka and karat sharing INDEL sites | 237,581 |
| Tongka SNP sites | 7,716,375 |
| karat SNP sites | 7,125,857 |
| Tongka and karat sharing SNP sites | 4,519,827 |


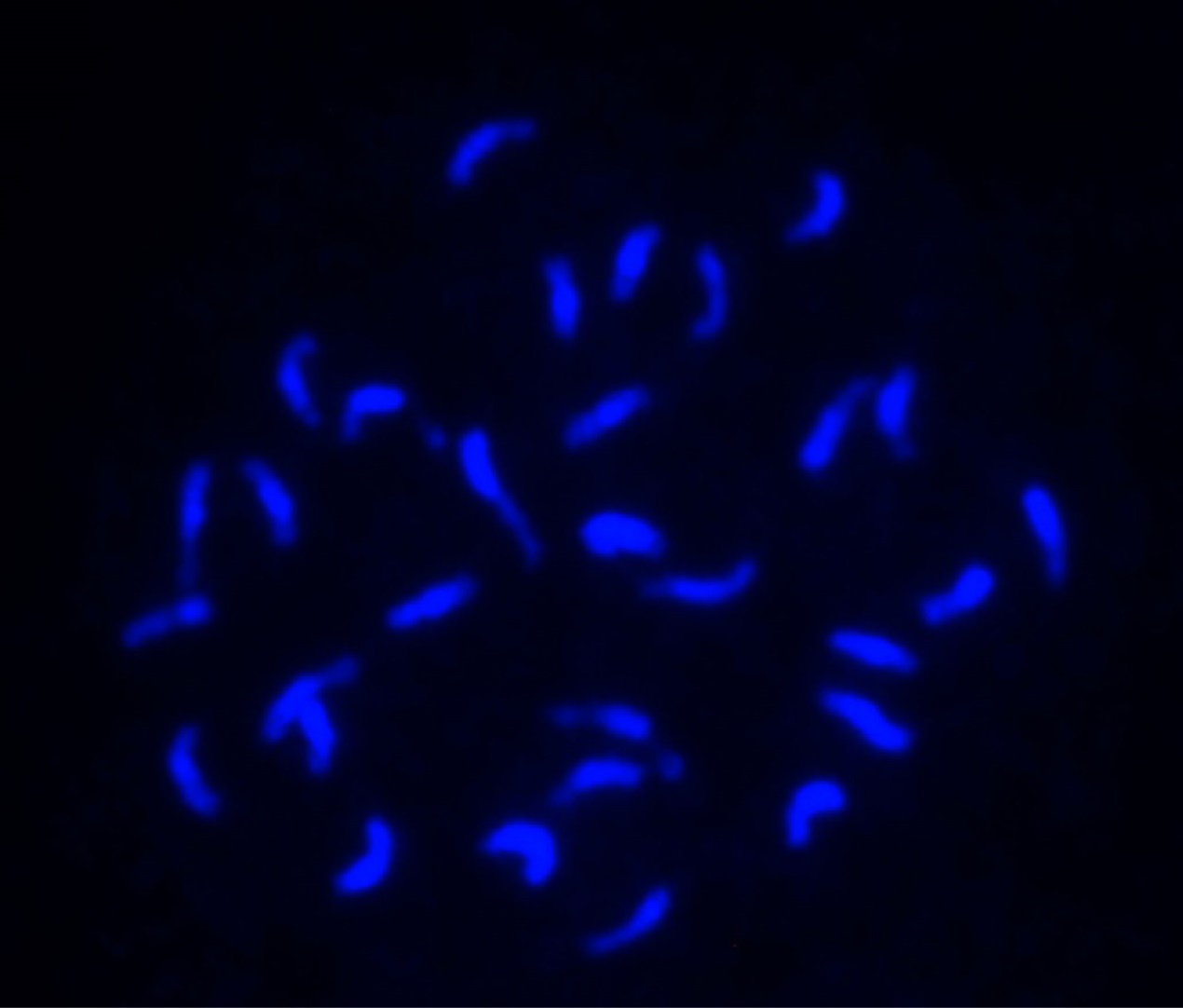


Figure S1 Fluorescent staining of chromosomes of *Musa troglodytarum L.* karat. The root tip of. karat plants staining with DPAI and screened under ultraviolet and fluorescence microscopy.


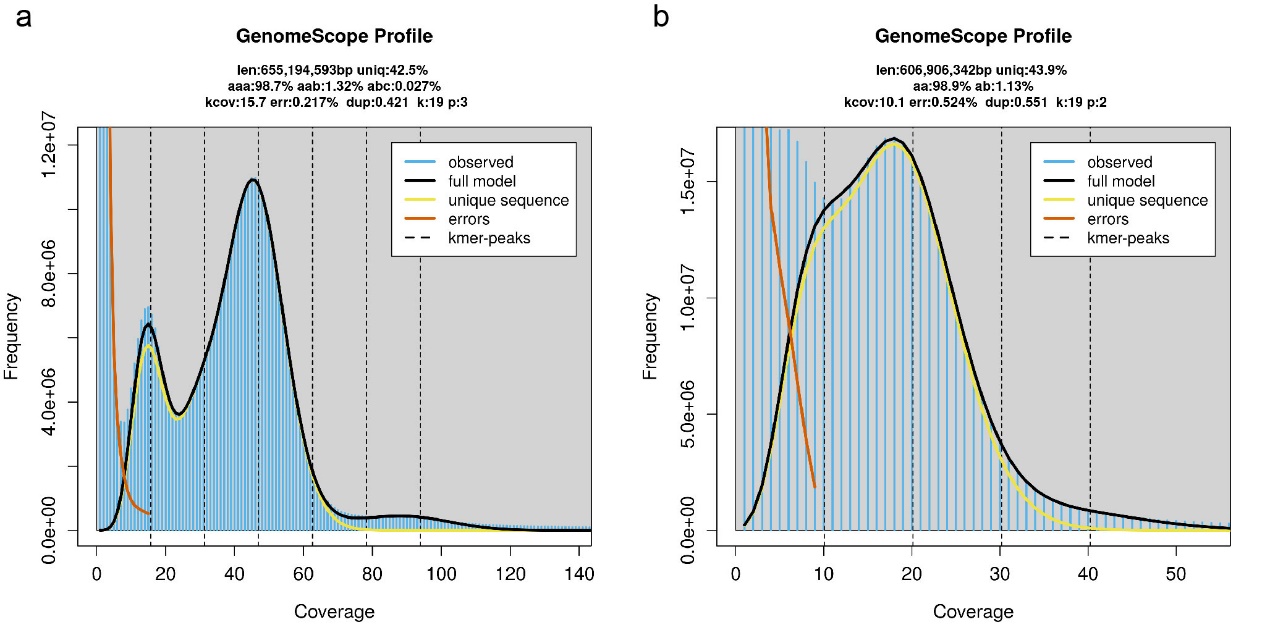


Figure S2 GenomeScope profile of karat and tongkat.


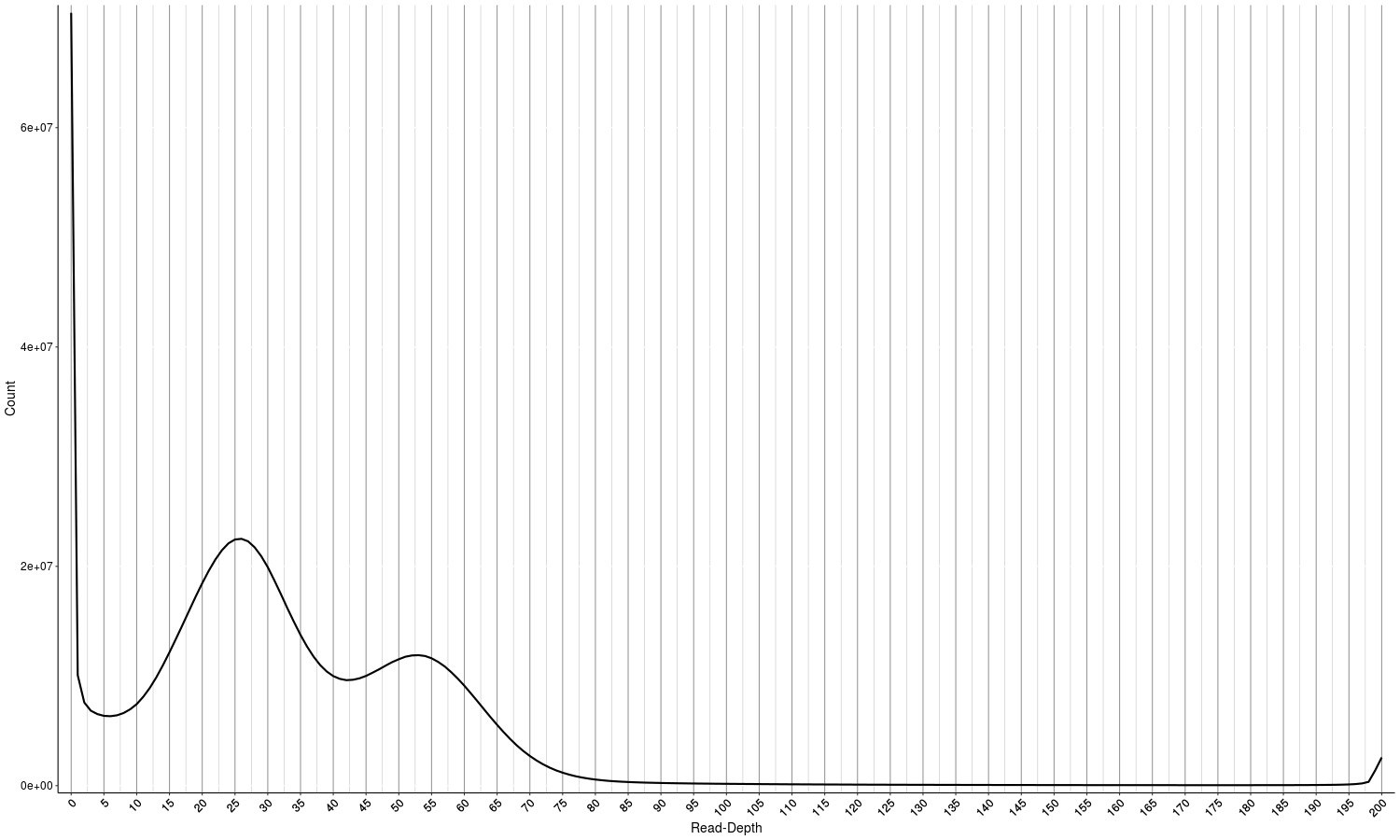


Figure S3 Bimodal histogram for Purge Haplotigs processing. The cutoff values for low, mid, and high points were 6,42 and 105, respectively.


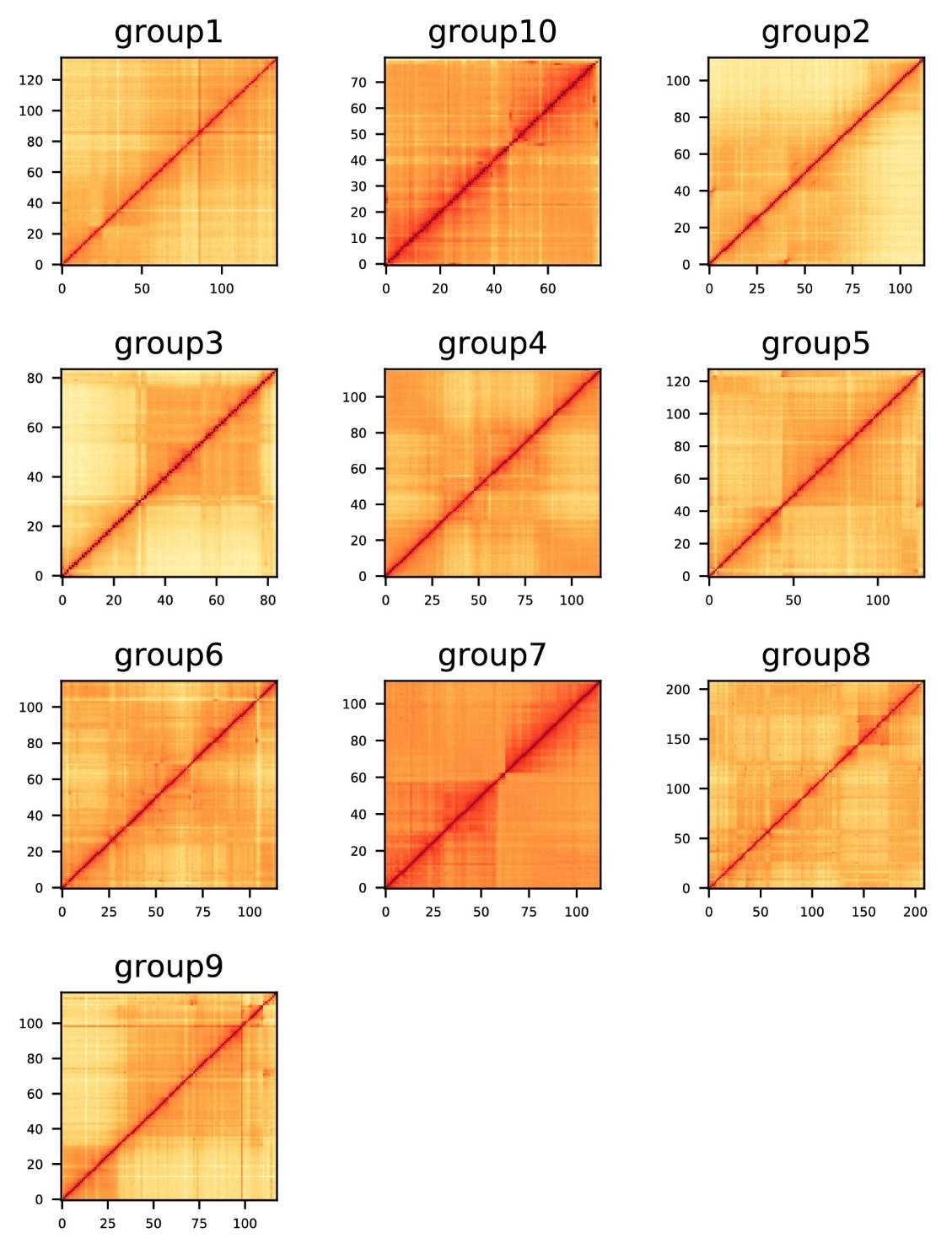


Figure S4 Hi-C mapping of chromosomes of the T genome.


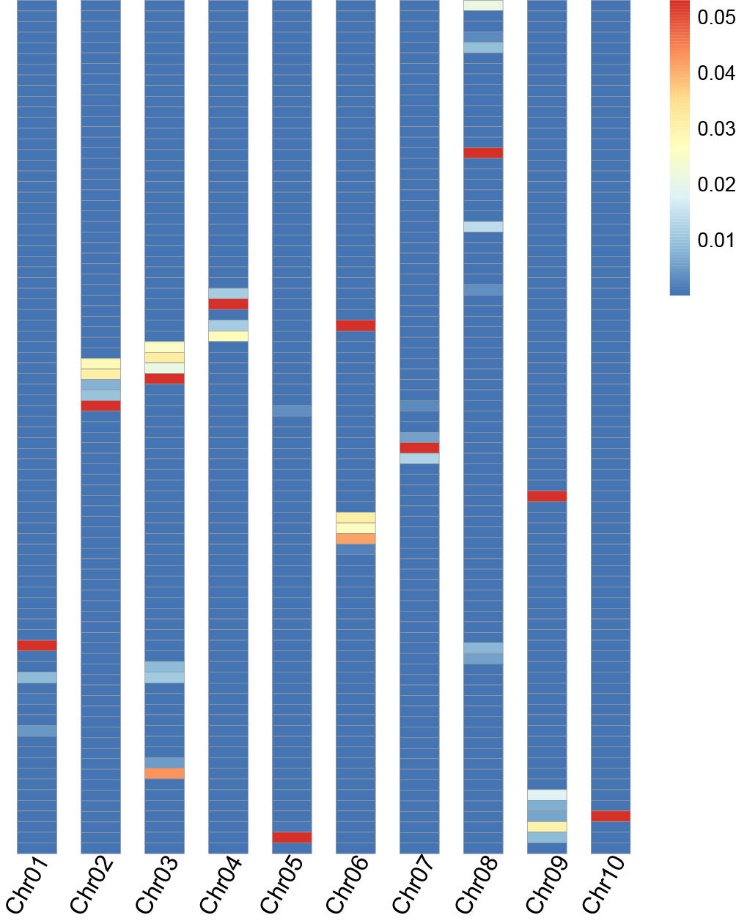


Figure S5 Heatmap of density of Nanica LINE.


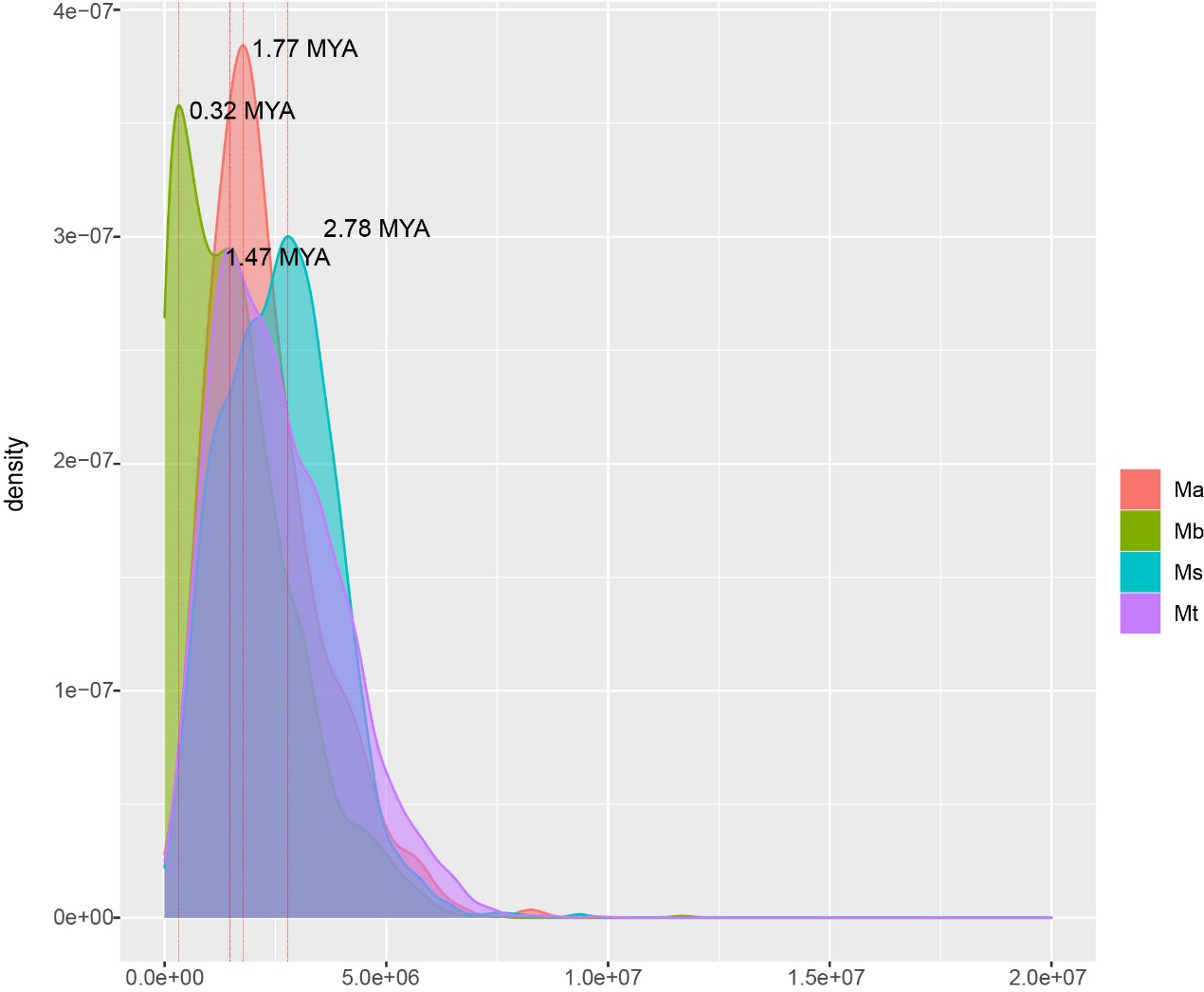


Figure S6 Analysis of LTR insertion times of the A, B, S and T genomes. Ma, *Musa acuminata;* Mb, *Musa balbisiana*; Ms, *Musa schizocarpa*; and Mt, *Musa troglodytarum.*


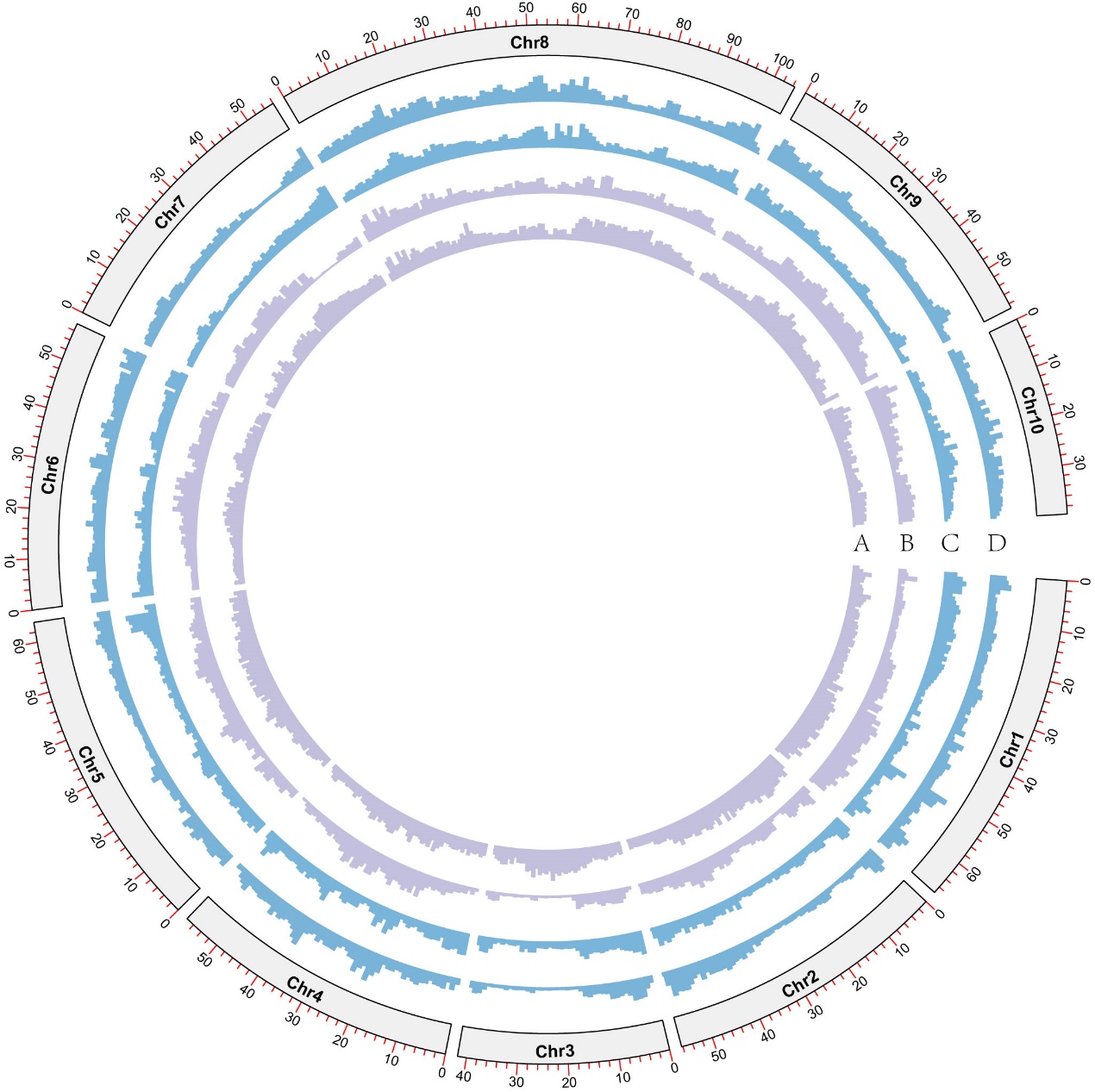


Figure S7 Distribution of SNP and indel sites in karat and tongkat. Indel sites in tongkat (A) and karat (B) and SNP sites in tongkat (C) and karat (D).


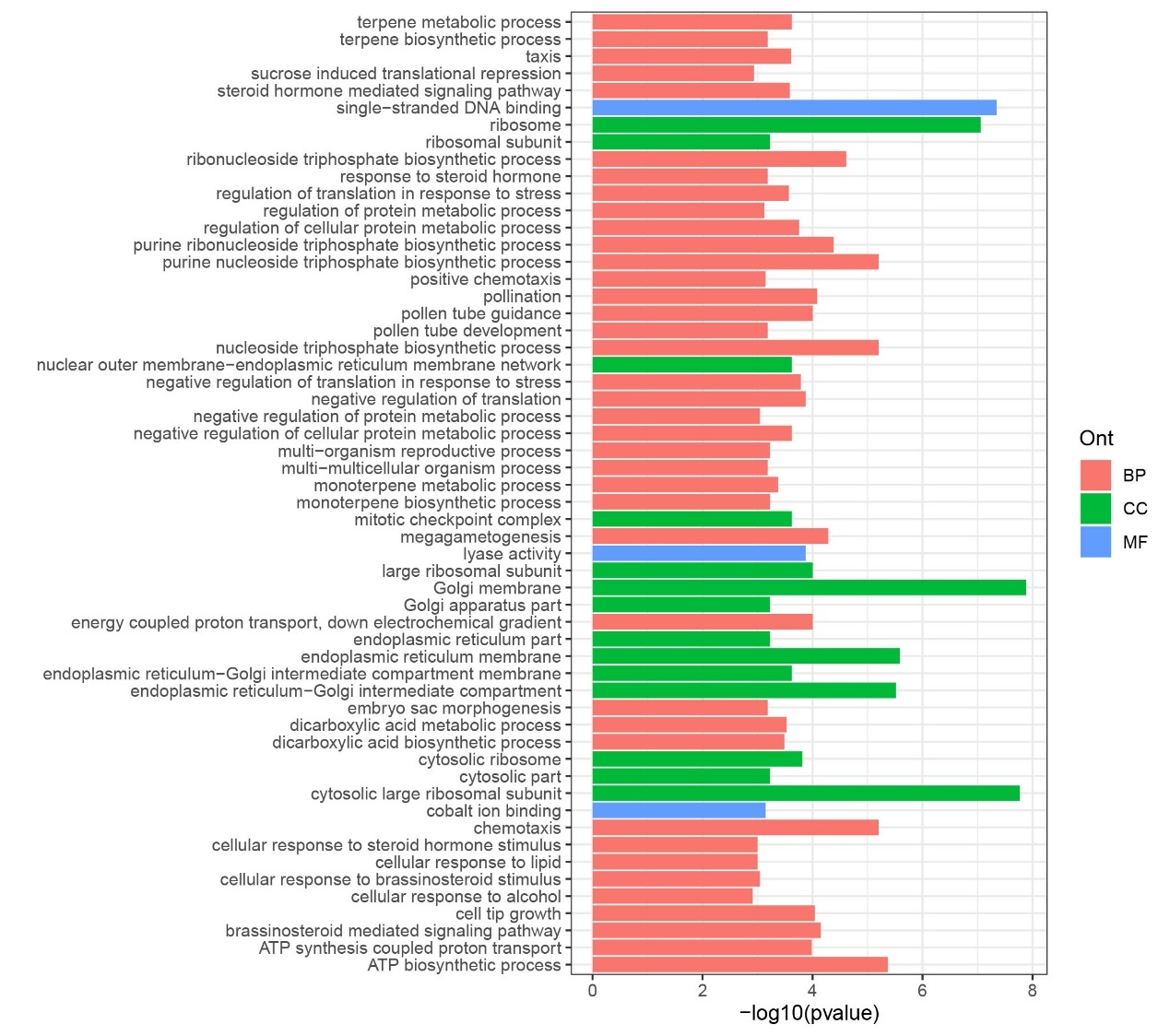


Figure S8 GO enrichment analysis of T genome specific genes.


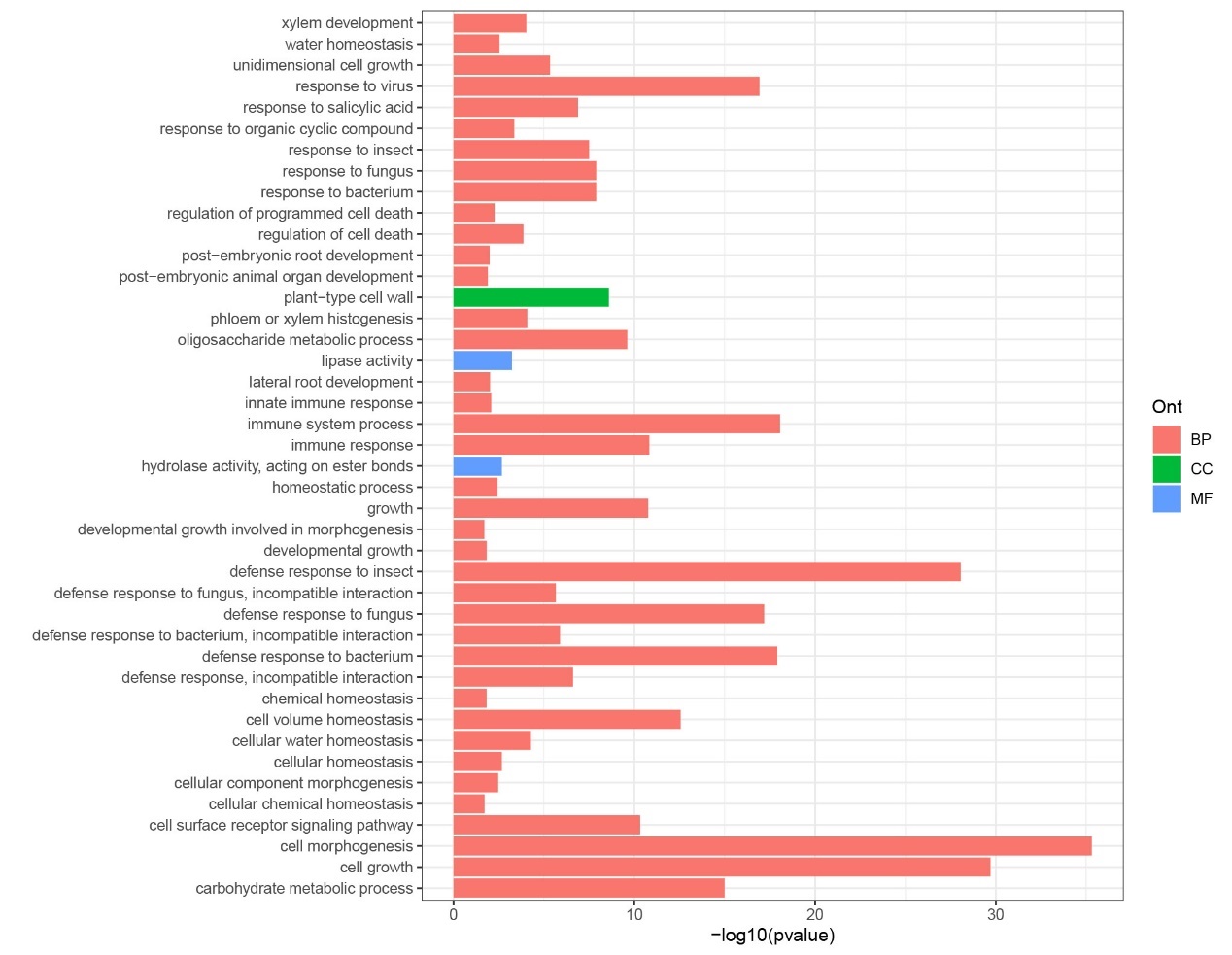


Figure S9 GO enrichment analysis rapidly evolving gene families in T genome.


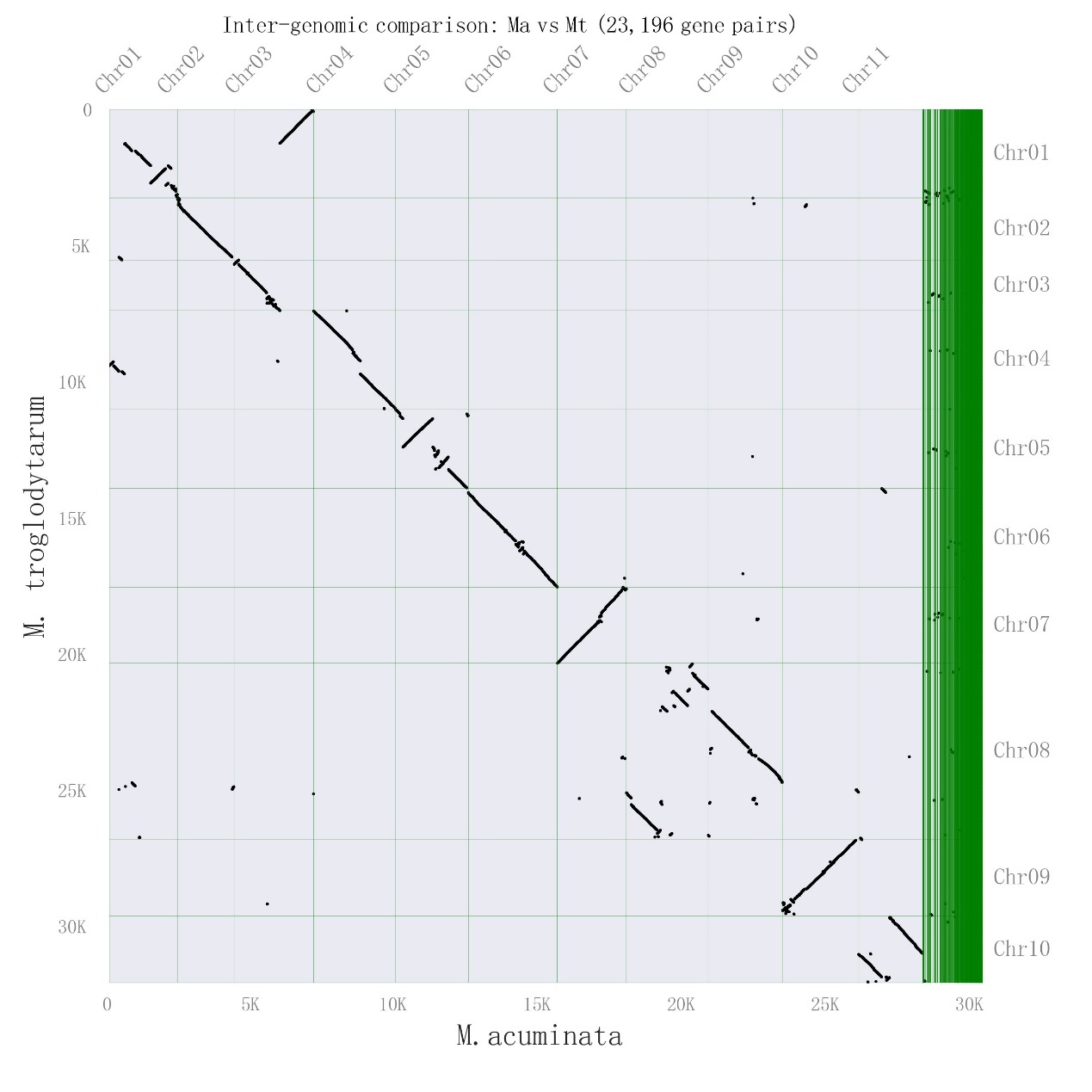


Figure S10 Dot plot of syntenic bocks between *M. troglodytarum* and *M. acuminata*.


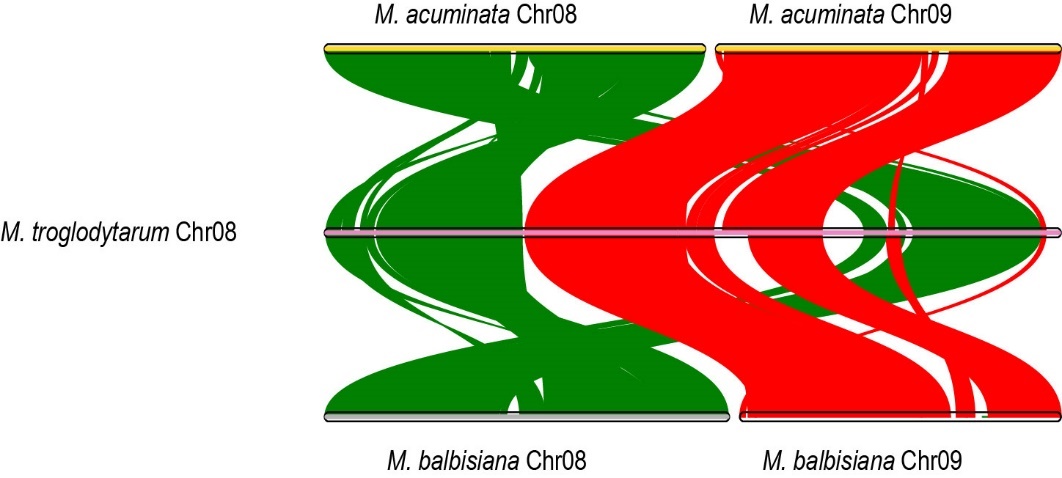


Figure S11 Synteny map of chromosome 8 and 9 among *M. troglodytarum*, *M. acuminata* and *M. balbisiana*.


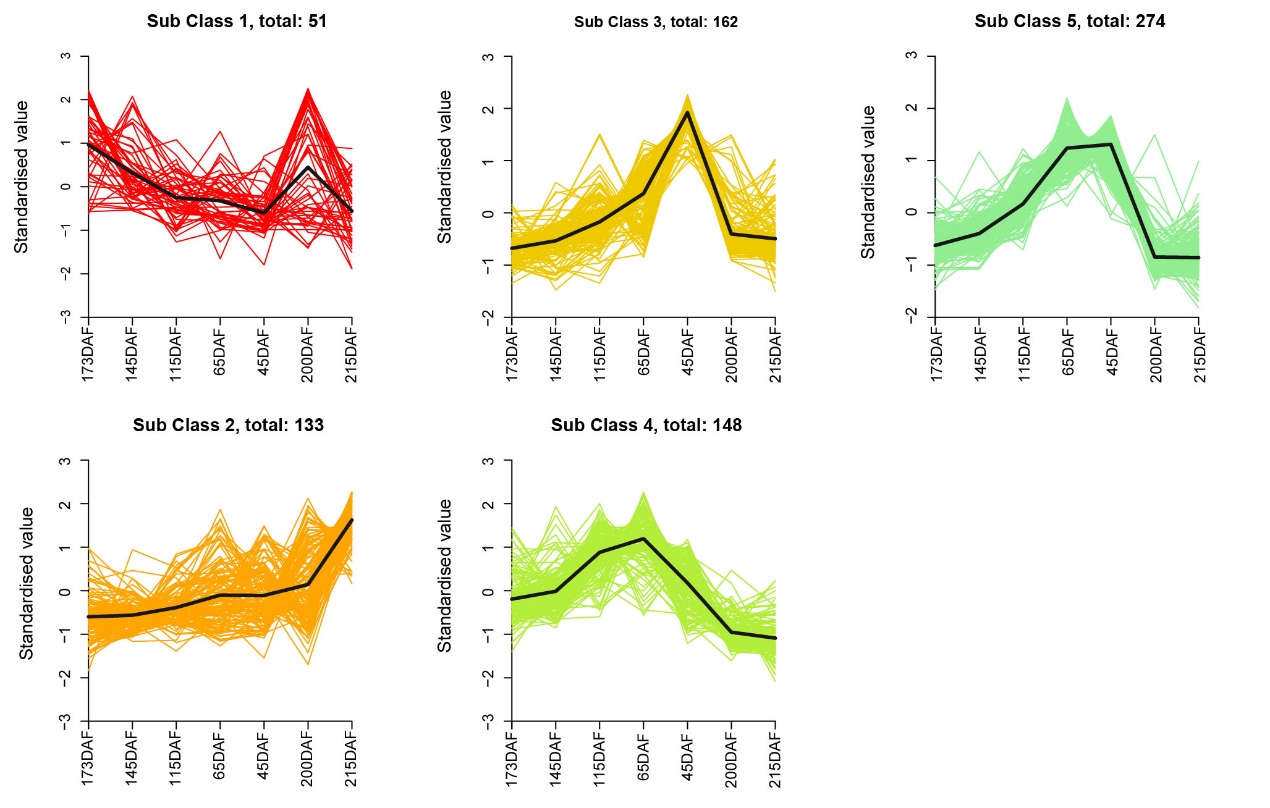


Figure S12 Clusters of metabolites in karat fruit pulp. DAF, days after flowering.


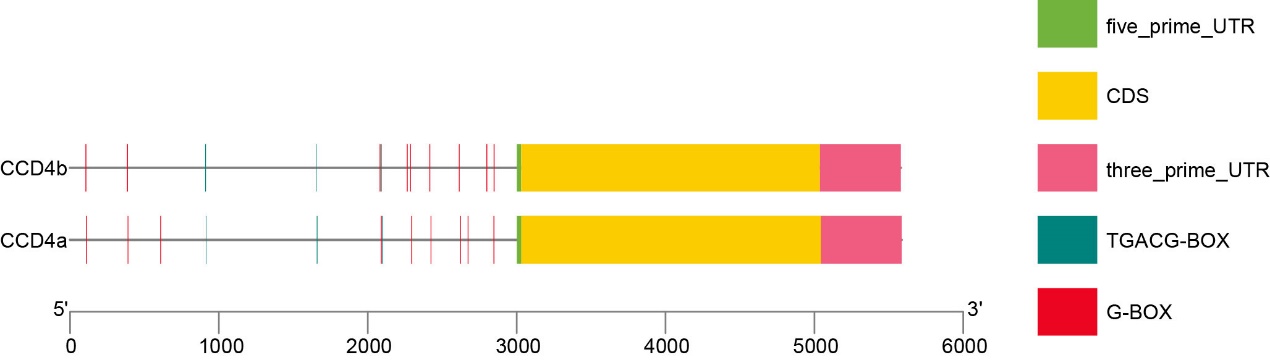


Figure S13 The distribution of JA response element G-box and TGACG-box motifs in the promoters of *MtCCD4s*.


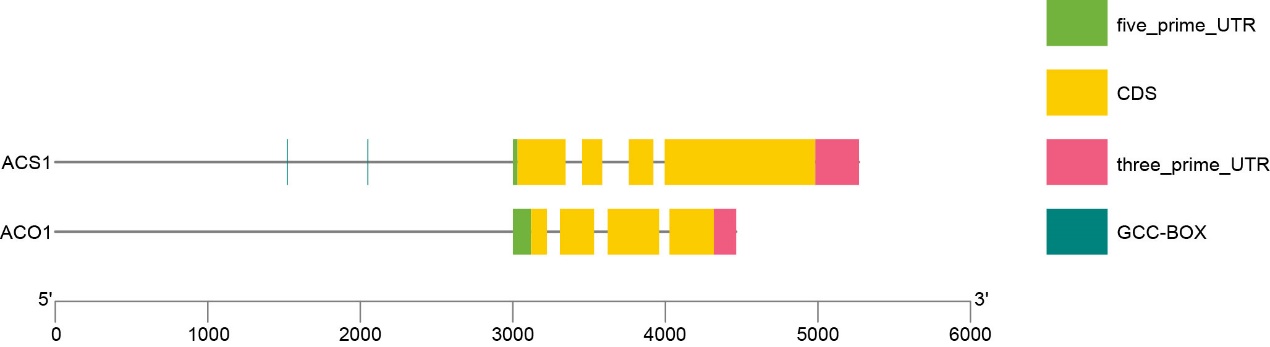


Figure S14 The distribution of GCC-boxes in the promoters of *MtACO1* and *MtACS1*.


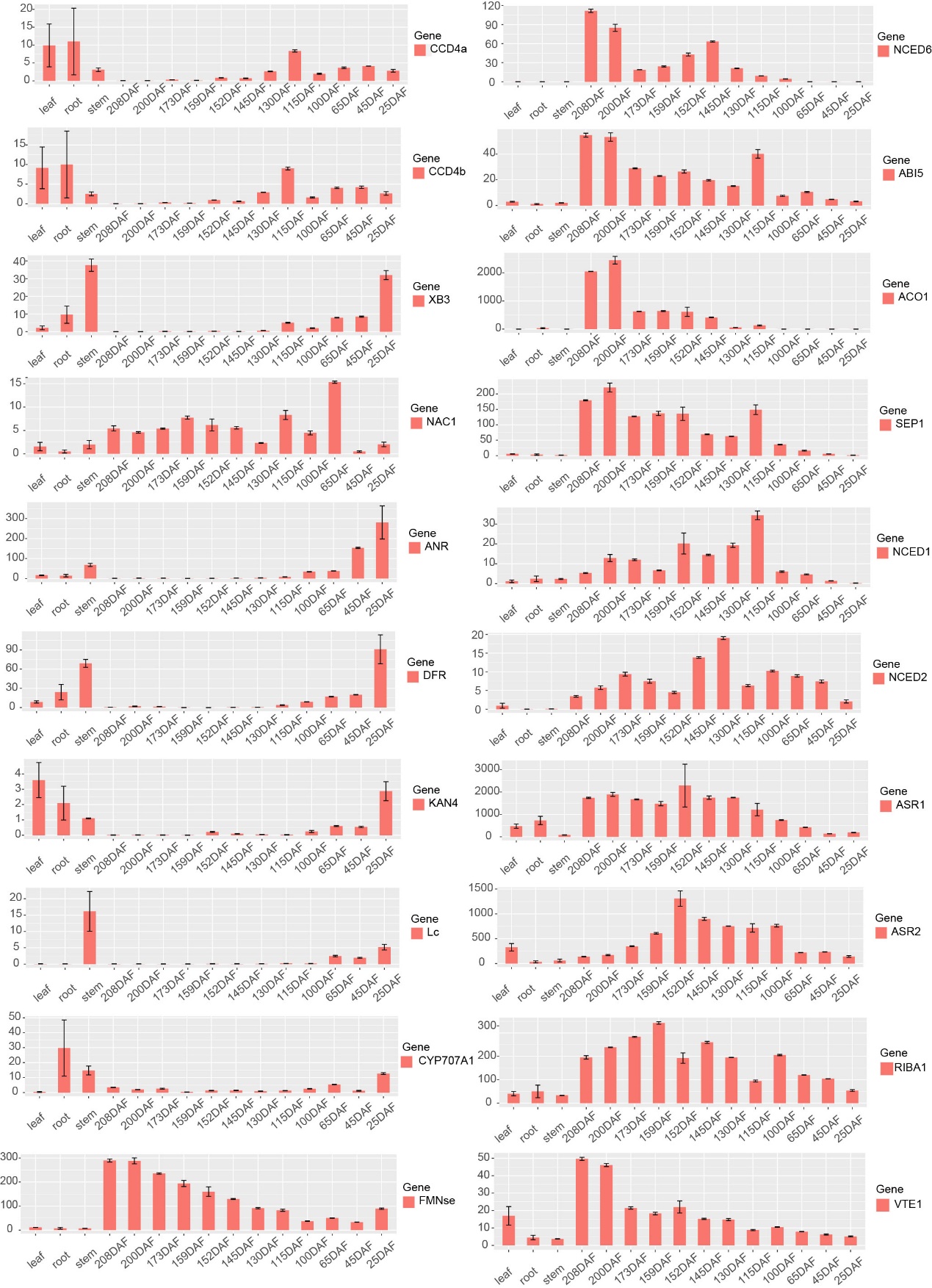


Figure S15 Expression patterns of genes involved in karat fruit ripening. Gene expression was normalized to FPKM (fragments per kilobase of transcript per million read pairs). DAF, days after flowering.


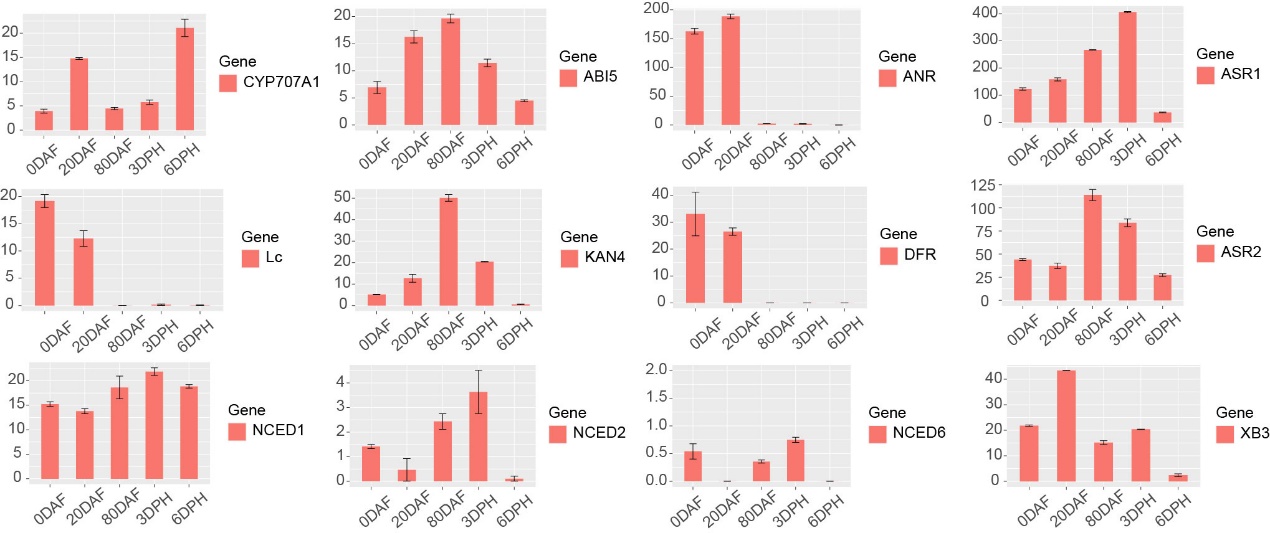


Figure S16 Expression patterns of genes involved in FJ fruit ripening. Gene expression was normalized to FPKM (fragments per kilobase of transcript per million read pairs). FJ (Fen jiao), dwarf banana. DAF, days after flowering. DPH, days post-harvest.


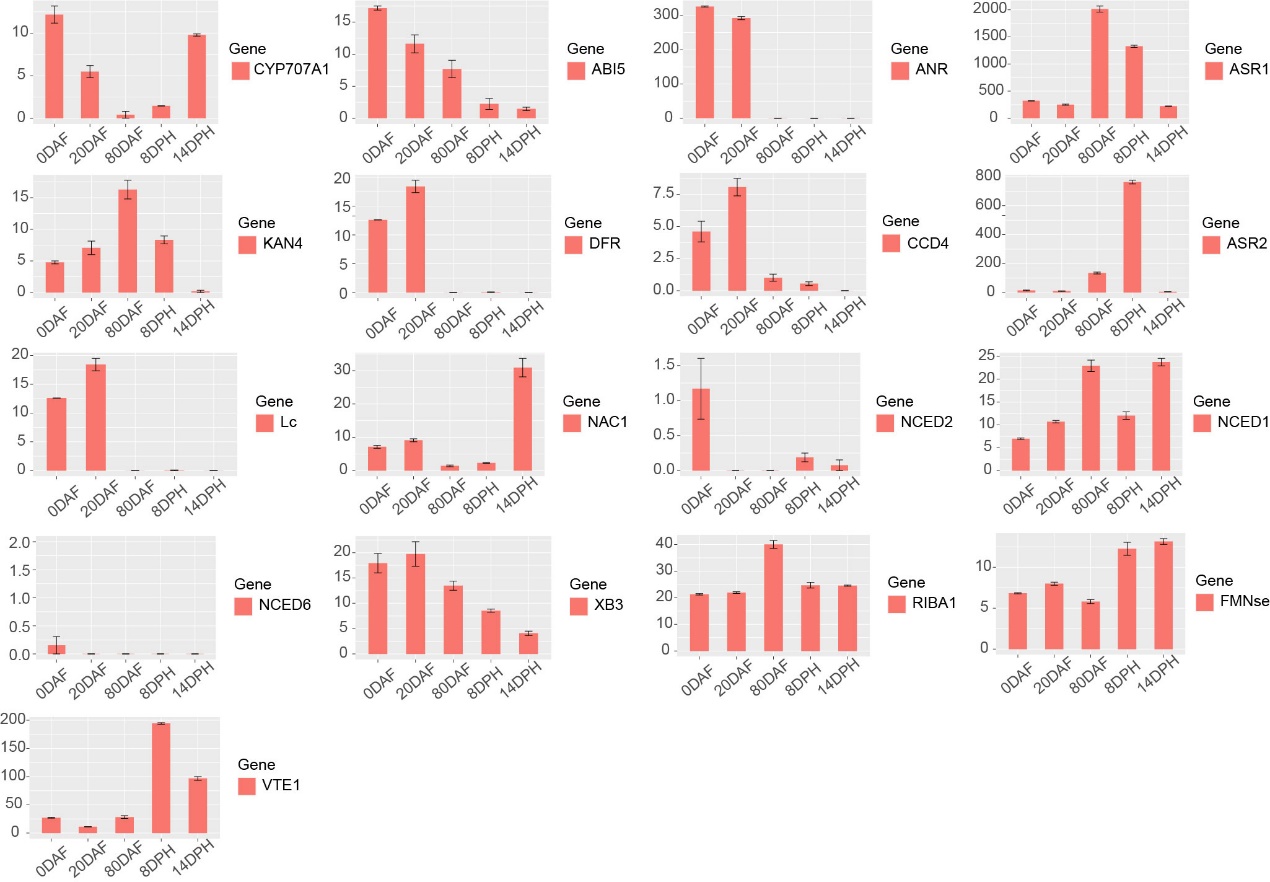


Figure S17 Expression patterns of genes involved in BXJ fruit ripening. Gene expression was normalized to FPKM (fragments per kilobase of transcript per million read pairs). BXJ (BaXi Jiao), Cavendish banana.


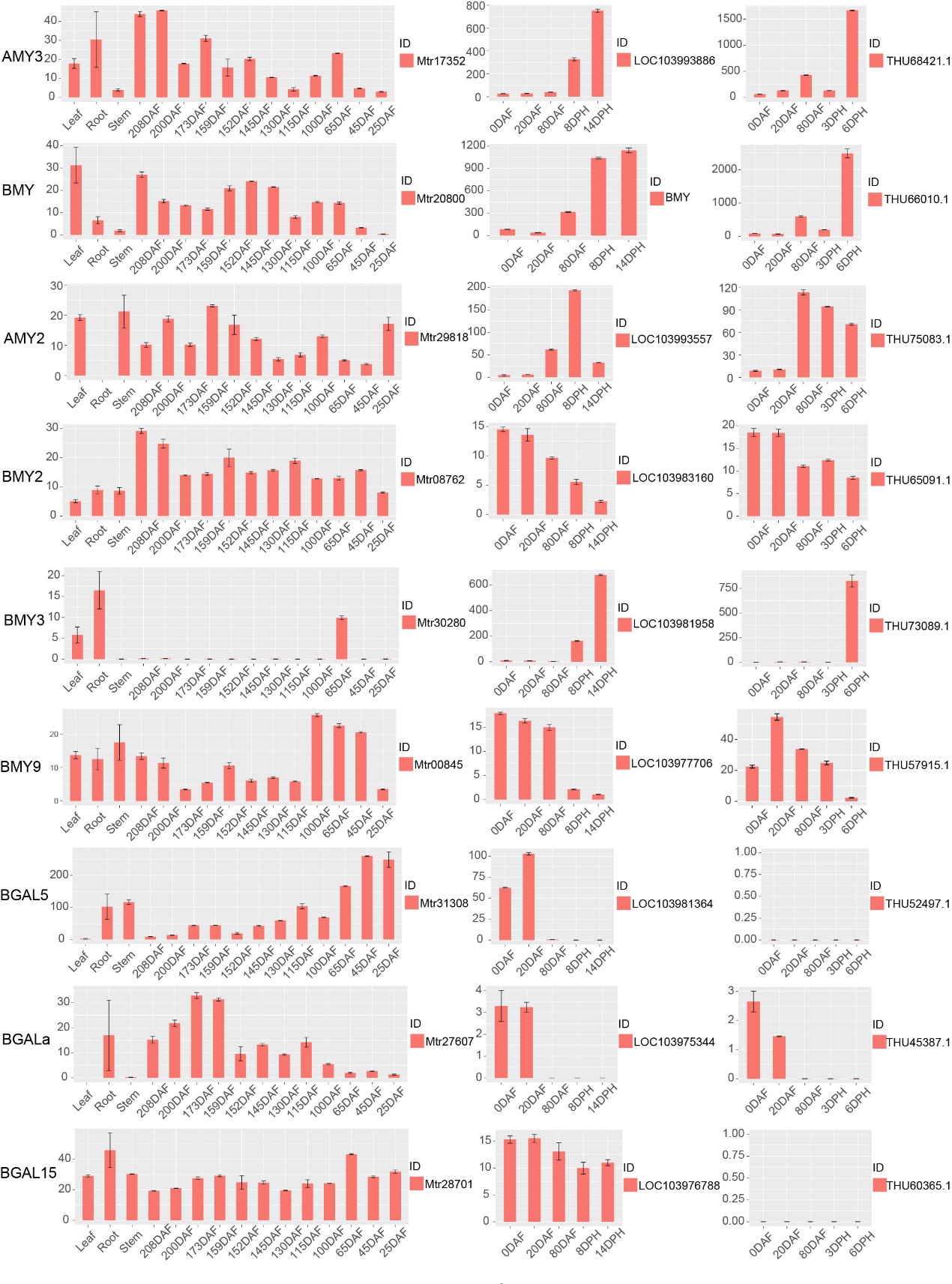

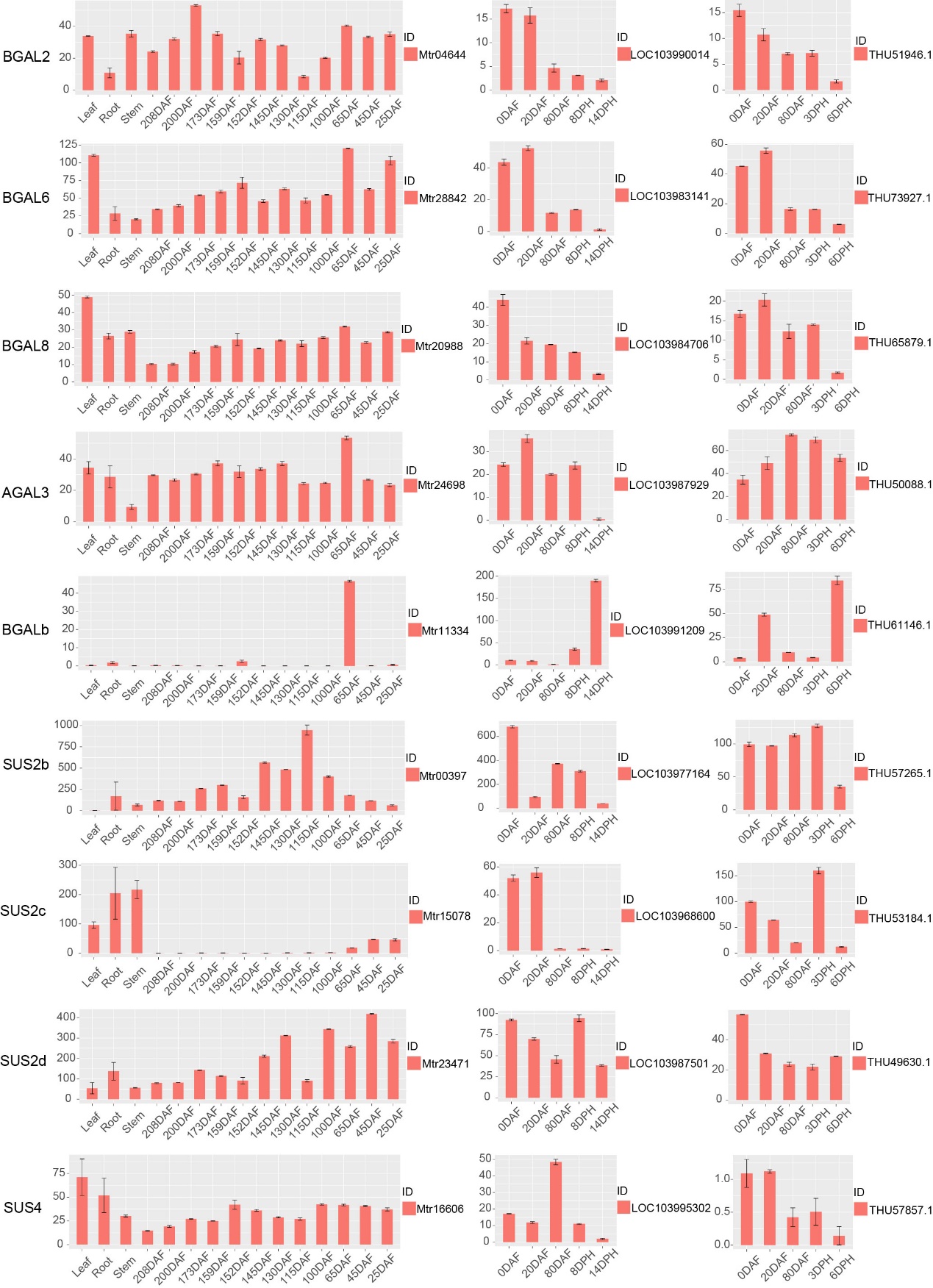


Figure S18 Expression patterns of genes involved in starch degradation and galactose accumulation. The gene IDs of M. *troglodytarum, M. acuminata and M. balbisiana* start with ‘Mt’, ‘LOC’ and ‘THU’, respectively. Gene expression was normalized to FPKM (fragments per kilobase of transcript per million read pairs).


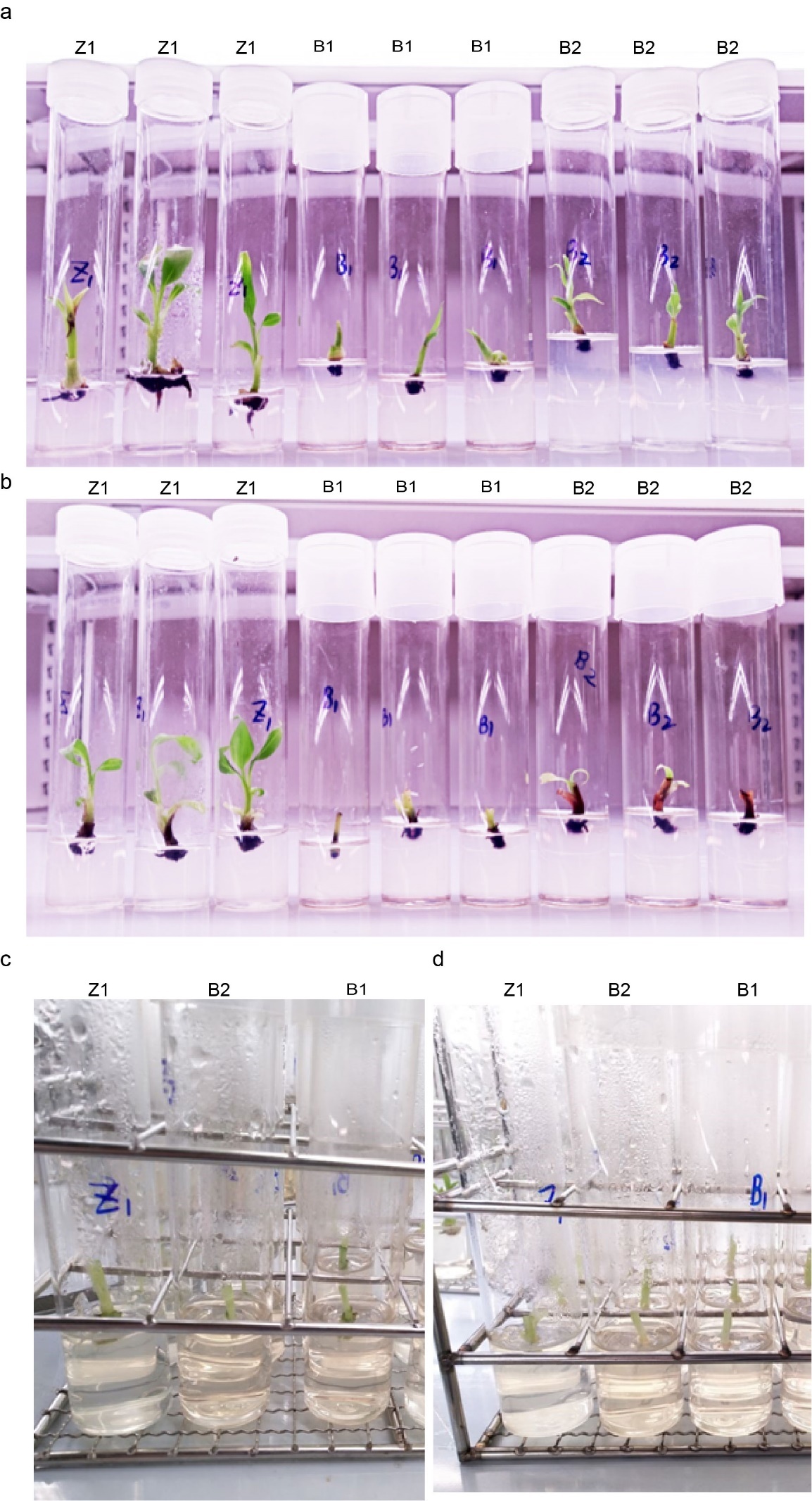


Figure S19 The effect of exogenous application free galactose on shoot buds of karat and BXJ. The shoot buds of karat (a) and BXJ (b) grown 20 days under a controlled environment (10 h light, 35% relative humidity and 25 °C). The karat (c)and BXJ (d) shoot buds (2-3 cm) were transferred into half-strength MS supplemented with Gal (10 mM, B2; and 100 mM, B1) and sucrose (10 mM, Z1), respectively. BXJ (BaXi Jiao), Cavendish banana.
